# Observational studies of exposure to tobacco and nicotine products: Best practices for maximizing statistical precision and accuracy

**DOI:** 10.1016/j.isci.2025.111985

**Published:** 2025-02-08

**Authors:** Gal Cohen, Steven Cook

**Affiliations:** 1 Rose Research Center, Raleigh, NC 27617, USA; 2 Epidemiology Department, University of Michigan School of Public Health, Ann Arbor, MI 48109, USA

**Keywords:** Health sciences, Medicine, Research methodology social sciences

## Abstract

Non-randomized observational studies can track risk-induction and -reduction associated with real-world use of non-combusted nicotine and tobacco products. The objective of this analysis was to evaluate the precision and accuracy of recent studies and to identify opportunities for further optimizing future study designs. The ROBINS framework for minimizing statistical bias was translated to specific considerations that spanned the selection and quantification of cohorts, exposure, and outcomes. These principles were then considered within the context of a recent comprehensive meta-analysis, representing 107 observational studies, which evaluated the effects of using electronic cigarettes (ECs), combusted cigarettes (CCs) and dual use of both. The meta-analysis had previously reported the relative risk from all-sources, including tobacco use and non–tobacco use. We now report the product use–specific risk associated with displacing CCs with ECs indicated from the primary references, along with observations regarding the precision of characterization of CC and EC exposure in the cited studies.

## Graphical abstract

**Figure:**
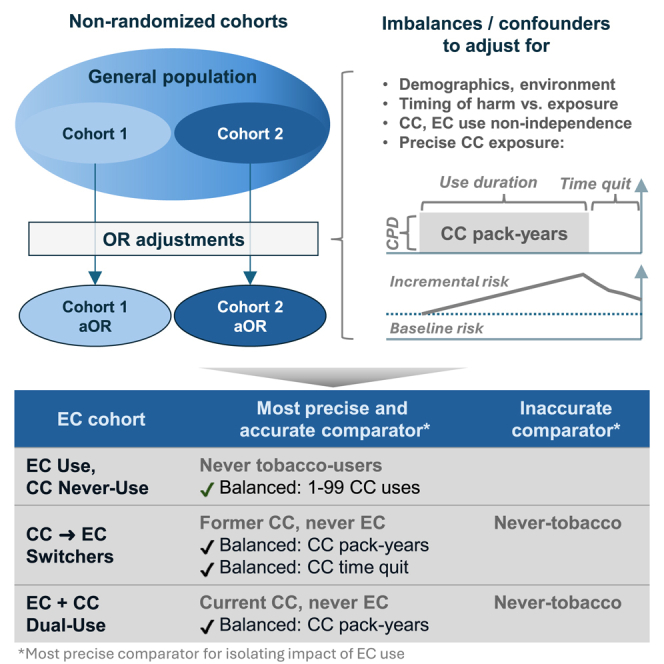

## Introduction

Smoking kills 480,000 people per year in the US alone.^1^ Pharmaceutical cessation products have been proven to help stop smoking, and yet, 28 million US adults still smoke, and global prevalence exceeds 1.3 billion.^2^^,^^3^ For adults who cannot or will not quit smoking, non-combusted tobacco and nicotine products were developed as potentially harm-reducing alternatives. Electronic cigarettes (ECs) are the most prevalent of these products, with use by 11–17 million adults in the US,^4^ and this study is focused on best practices for evaluating the risk-inducing and risk-reducing health effects of EC use. However, the principles discussed here are broadly applicable to other tobacco and nicotine products.

ECs have been widely available for over a decade. Major cross-sectional and longitudinal studies, including the Population Assessment of Tobacco and Health Study (PATH), Behavioral Risk Factor Surveillance System (BRFSS), National Health Interview Survey (NHIS), and National Health and Nutrition Examination Survey (NHANES), collect an ever-growing body of nationally representative data about the health effects associated with real-world EC use patterns.^5^^,^^6^^,^^7^ By analyzing these databases, non-randomized observational studies of ECs can evaluate incremental risk (vs. non-use), relative risk vs. CC (combusted cigarettes), risk reversal (associated with switching away from CCs), and the risk associated with dual use of ECs and CCs.

## Methods

This article was informed by the Risk Of Bias In Non-randomized Studies (ROBINS) framework, developed through expert consensus to evaluate and mitigate statistical bias in non-randomized studies.^8^^,^^9^ The principles from the ROBINS framework, as translated to the study of tobacco and nicotine products, were then applied to a recent meta-analysis, chosen because it represented possibly the most extensive recent meta-analysis in the field, spanning major disease categories.^10^ The draft results were posted on a preprint server to allow for open peer review and feedback.^11^

## Precision of sample definition, exposure characterization, and outcome metrics impact study accuracy

**Figure 1:**
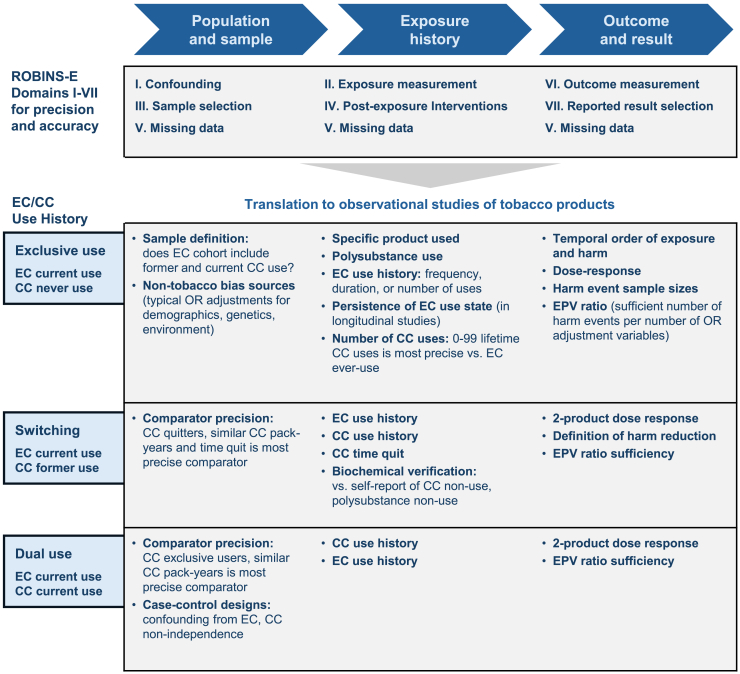
Factors impacting precision and accuracy of observational studies of exposure to electronic cigarettes EC (electronic cigarette); CC (combusted cigarette); OR (odds ratio). ROBINS-E domains aggregate into three categories, spanning characterization of population and sampled cohorts, exposure history, and outcomes and results. Observational studies of EC use may include one or more of the cohorts represented across the three rows: EC users who are never, former, and current (dual) users of CC. The factors here are described specifically for exposure to EC and CC, but can generalize to other tobacco and nicotine products (see also Table S1.1).

The ROBINS framework identifies seven domains that can impact precision and accuracy of observational studies. Broadly, these sources of potential bias pertain to three groupings: definitions of population and sample, characterization of exposure history, and selection of outcomes and results, as enumerated in Figure 1. To translate the ROBINS-E framework to studies of tobacco and nicotine products, considerations of particular impact and relevance are also outlined in Figure 1. This review primarily discusses examples pertaining to the assessment of ECs, but the concepts are broadly generalizable to other categories of nicotine substitution products, such as heated tobacco products (HTPs) and oral nicotine products (ONPs).

Attaining a high degree of accuracy and precision can be challenging because non-randomized studies, by definition, compare outcomes across heterogeneous cohorts which may differ in demographic, environmental and background risk factors. Therefore, unadjusted odds ratios (ORs) are typically adjusted (aOR) to address these potential confounds. However, beyond these typical adjustments, evaluation of the impact of tobacco product use involves disambiguating between multiple sources of exposure (EC, CC, and non-tobacco sources of risk) across populations which span different histories of product use. A study subject can be a never-, former- or current-user of a given product, and their use state can transition over time.^12^^,^^13^ The harm associated with each product (EC, CC) is dependent on cumulative use history (dose per use, frequency of use, duration and timing of use, and quit duration). Capturing the comprehensive use history of these tobacco products, along with dose-response validation, can help to ensure precision and accuracy of assessments.^14^

### Precision of cohort definitions impacts study accuracy

#### Selection of exposure cohorts and comparators

Samples termed ‘EC users’ often include individuals with histories of CC former and current use (switching and dual-use, respectively). Data from Wave 6 of the PATH study provides an example. Among older (35+ years old) current EC users, 91% were current or former CC users. Older current EC users who were never CC users (<100 lifetime cigarettes smoked) only represented ∼80 participants in this survey of over 30,000 participants (see Figure S1-1).

This multi-source exposure history may be particularly subject to confounding. For instance, common liability for CC and EC use, use of EC for switching or cessation, and reverse causation (switching to EC use after a diagnosis or concern of harm from CC use) can cause spurious positive associations of risk if not carefully adjusted for.^15^^,^^16^ CDC data (2021 NHIS survey) illustrates that current EC users were 2.7x more likely than current EC non-users to be current CC users and 1.8x more likely to be former CC users, while 55% less likely to be never CC users (see Table S1.2).^17^ EC use frequency is also elevated among people who have a history of smoking more CC.^18^

At the same time, multiple studies have reported that frequency of CC use is negatively associated with EC use, when EC are used for quitting cigarettes.^11^^,^^19^^,^^20^^,^^21^ Therefore, EC and CC exposure patterns should not be assumed to be independent of each other.

As described in the Graphical Abstract and Table S1.2, because of the lack of independence between EC and CC use, it is important to segment into parallel EC populations based on CC exposure (never CC, former CC, and dual-use) rather than stratifying into samples that have experienced harm vs. no harm (i.e., avoid case-control approaches). The case study discussed below (Glantz et al., 2024) includes two cautionary examples which selected case-control designs and subsequently reported that CC use was not associated with increased risk of cardiovascular harm (with former CC use being associated with reduced risk in one study). In other words, they attributed the entirety of risk associated with tobacco product use to EC use.^10^^,^^22^^,^^23^

Appropriate comparator cohorts are also important for accuracy and precision. Switching from CC to EC is most precisely compared to former CC smoking, and dual-use is most precisely comparable to exclusive CC smoking. Furthermore, as discussed in the next section, odds adjustments should account for imbalances in CC exposure between the EC cohorts and comparator CC cohorts.

### Precision of exposure characterization impacts study accuracy

#### Specific EC product used

**Figure 2:**
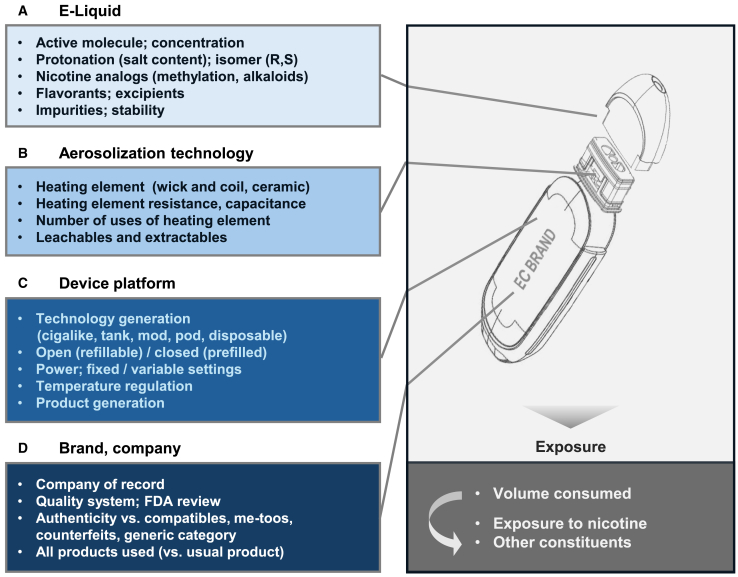
EC product characteristics impacting exposure Product characteristics which can impact exposure include: (A) the e-liquid composition, (B) the aerosolization technology, (C) the device platform, and (D) the brand and company of record. Schematic diagram adapted from patent assigned to Shenzhen Smoore Technology, Ltd.^24^ This platform and similar variants are incorporated in the NJOY Ace (Altria, Richmond, VA) and VUSE Alto (R.J. Reynolds, Winston-Salem, NC), which are FDA-authorized, later-generation, reusable closed-system, nicotine-salt (1.8%–5.0%) pod products with fixed power settings and higher-capacitance ceramic heating elements which are replaced with each pod (1.8mL–1.9mL e-liquid per pod). See also Document S1.

Electronic cigarettes represent a heterogeneous and evolving category. Exposure to nicotine and toxicants, as well as efficacy in displacing CC, may vary dramatically across products. Therefore, specification of which products were used can be an important consideration in the precision and generalizability of any results. Factors impacting EC exposure include e-liquid and nicotine composition, aerosolization technology, device platform, specific brand and FDA verification of manufacturing quality systems of the company of record (see Figure 2 and Document S1 for additional details).

#### Comprehensive CC use history

**Figure 3:**
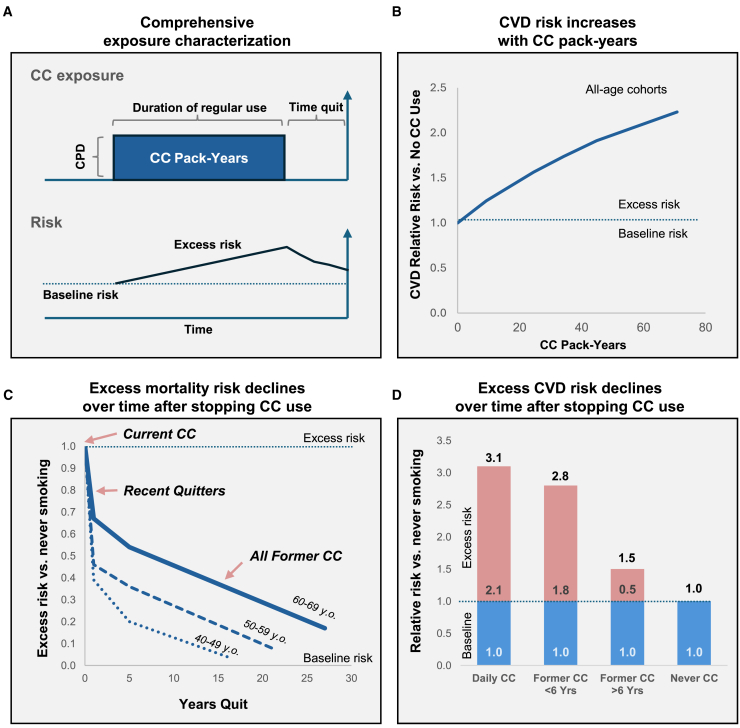
Comprehensive characterization of CC exposure includes intensity, duration and timing of use CPD (cigarettes per day; smoking intensity); CVD (cardiovascular disease); CC (combusted cigarettes); YO (years old). (A) A comprehensive characterization of CC exposure includes smoking intensity (CPD, or cigarettes per day), duration of regular use, and time quit (if relevant). Top graph illustrates CC smoking intensity (Y axis) vs. time (X axis). Bottom graph illustrates corresponding risk (Y axis) vs. time (X axis). (B) CVD risk (Y axis) vs. CC pack-years (X axis). Redrawn from (Lubin et al., 2016).^25^ (C) Excess mortality risk due to CC use (total risk - baseline risk; Y axis) vs. time quit (X axis). Redrawn from (Cho et al., 2024).^28^ (D) CVD risk (Y axis) vs. CC use history status (X axis). Redrawn from (Farsalinos et al., 2019).^29^

Next, across all tobacco products, risk is typically dependent on both frequency and duration of use (see Figure 3, panel A). The Atherosclerosis Risk in Communities (ARIC) prospective multi-decade longitudinal study demonstrated that cardiovascular disease (CVD) risk was primarily determined by cigarette pack-years, with duration of smoking being particularly impactful of risk (see Figure 3, panel B).^25^ Similarly, for chronic obstructive pulmonary disease (COPD), duration of smoking is more predictive of risk than cigarettes per day.^26^ In summary, while in many instances it may be sufficient to characterize CC exposure dose by a single pack-years metric, characterizing both duration of use and cigarettes per day may increase statistical precision of the exposure characterization and its resultant impact on health risks.^27^

Characterization of the recency of discontinued CC use also is important for analytic precision. As shown in Figure 3 (panel C), mortality risk is elevated in people who smoke. After quitting smoking, risk trends downwards over a course of years and decades, with risk decreasing more slowly with older people who smoke, possibly due to more pack-years of harm accumulation.^28^^,^^30^ Figure 3 (panel D) shows that CVD risk is markedly lower in those who stopped CC smoking more than six years previously compared to those who are more recent quitters.^29^ Several other studies have also reported that CVD risk declines over a time course of decades after stopping smoking.^25^^,^^31^ Likewise, for COPD, model accuracy increased when time since quitting CC was incorporated as a predictive factor.^32^ However, in analyses of cohorts which had on average stopped smoking a decade or more earlier, time since stopping smoking was less predictive for CVD risk than other metrics such as cigarettes per day.^31^^,^^33^

Therefore, EC switching impact should be measured against the maximum harm reversal which would be possible in that time-period, for instance if quitting with traditional pharmaceutical approaches or with abstinence (“cold turkey”). As time since switching from CC to EC increases from months to years, the upper limit for risk reversal increases from zero to approaching the level of never smoking. Consequently, when calculating aOR among current EC users who formerly smoked CC, the correct OR adjustment comparator is not necessarily the typical ex CC-user, who may have quit a decade or more ago, but rather a cohort of ex CC-users, with similar CC pack-years, which has quit on average as recently as the EC sample (see also Graphical Abstract and Figure 1).

#### Comprehensive EC use history

For quantifying EC exposure, there is no standardized equivalent to the CC pack-years metric. Past 30-day frequency of use data may not be representative of cumulative exposure. Concerningly, most major national surveys, including BRFSS, NHIS, and NHANES, do not incorporate questions about duration of EC use. Without accounting for the frequency and duration of EC use, studies are limited in their precision and accuracy of measuring the impact of EC exposure; to assess the impact of EC exposure, it is imperative to consider this information.

**Figure 4:**
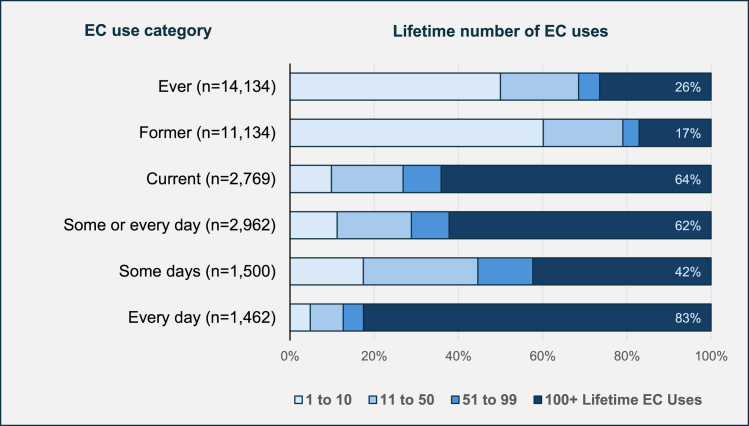
Lifetime number of EC uses, stratified by EC use category (PATH, Wave 6) PATH Wave 6 unweighted adult EC use data is illustrated. For each cohort, the proportion of the sample which has reported lifetime uses of EC numbering 1 to 10, 11 to 50, 51 to 99, and 100+ times is color-coded. Note: Current use (past 30-day use) sample size does not exactly correspond to the sum of current some-day and current every-day use, and ever-use does not exactly correspond to the sum of former and current use, because of inconsistencies in participant answers across different questions (see also Figure S1.1).

PATH does include time of first ever- or regular-use of EC, as well as lifetime number of uses.^6^ Figure 4 illustrates lifetime number of uses of EC for the following categories in the Wave 6 database: EC ever-use, former use, current use, use some days or every day, use some days, and use every day. Of the EC ever-use sample, 50% reported 10 or fewer lifetime EC uses, 23% reported 11 to 99 uses, and 26% reported ever-use of 100+ times. In other words, 74% of this cohort would not meet the threshold of “ever-use” as typically applied to CC use. Likewise, 58% of current some-day users reported fewer than 100 lifetime EC uses, while 83% of current every-day users reported 100+ lifetime EC uses.

#### Self-report vs. biochemical verification of (non)-exposure to nicotine and other substances

Self-reported metrics of exposure and harm are most accurate when verified through additional means. For instance, in a recent large randomized trial of EC and nicotine replacement therapy (NRT) for smoking cessation, 26% of self-reports of 7-day cigarette abstinence conflicted with exhaled CO (carbon monoxide) measurements in the EC group, and 33% of abstinence self-reports conflicted in the NRT group.^34^ COHb in blood samples can indicate daily exposure to combustion products, although it is non-specific for cannabis smoking as well and is not sufficiently sensitive to detect non-daily use. The hemoglobin adduct CeVal has a longer half-life and is thus a more sensitive blood-based biomarker of CC use.^35^ Relevant urinary biomarkers selective for CC use include the tobacco-specific nitrosamine NNAL (also found in smokeless tobacco), and 2CyEMA (indicative of acrylonitrile exposure from cigarette smoke).^36^^,^^37^ Propylene glycol (PG) has been proposed as a potential marker for EC use. However, in the observational setting it may not be specific enough vs. background dietary PG sources to discriminate between EC use vs. non-use for pod users of higher nicotine concentrations, who may consume a fraction of a mL of e-liquid per day (see Document S1 for more details).^38^^,^^39^^,^^40^

Given the increasing evidence base for cardiovascular and other harms associated with cannabis and alcohol, high prevalence of use, and propensity for multi-substance use, it is also important to adjust for the differential use patterns of these and other substances across study cohorts.^41^^,^^42^ In 2023, among US 12th graders, the incidence of current cannabis and alcohol use were 18.4% and 24.3%, respectively, relative to 16.9% for nicotine EC use. The incidence of daily use of cannabis and binge drinking were 6.5% and 10.2% respectively, relative to 5.8% for daily nicotine EC use.^43^ Because cannabis can be vaped, it is important to distinguish between nicotine and cannabis EC use in self-reports. Likewise, biochemical verification of cannabis and other drug use and non-use would be valuable in the PATH study; these data were sorely lacking during the EVALI wave which arose due to adulterated cannabis vapes; self-report of cannabis and other drug use can be unreliable.^44^

#### Verification of continuity of use state in prospective studies

Prospective studies typically segment cohorts based on their product use state at the start of the study. Over the course of the prospective period, participants may continue their product use, stop use, and/or transition to other products. A precise assessment of exposure should capture these ongoing product use patterns, in addition to current and past use, as they may exacerbate or ameliorate risk.

### Precision of outcome metrics impacts study accuracy

#### Temporal order of exposure and harm outcome

Exposure can only be causal for harm events that occur after the exposure. Consequently, retrospective studies should carefully evaluate the timing and duration of exposure, and relative timing at which harm events occurred. For example, PATH Wave 1 (fielded in 2013-14) contains a sample of *n* = 1,684 individuals who reported COPD (‘ever in life’). Of these, 1,252 (74%) reported that the harm event occurred 4 or more years earlier, meaning that they had the outcome before EC were widely available in the US marketplace.

#### Sufficient duration of product use for measured outcome; early markers of harm

Exposure should be of sufficient dose and duration to plausibly drive the underlying physiological disease process. For assessment of relative risk of exclusive use of EC vs. CC use or vs. non-use, for some databases or endpoints, EC usage data may not be of sufficient duration for precise analysis.^45^ For instance, COPD is most typically seen after 40 or more pack-years of smoking. Criteria for diagnosis of early COPD are in development, but an exemplar assessment framework still included a minimum of 10 pack-years of CC use.^46^ Generalized indicators of health may be predictive of earlier chronic changes in pulmonary function, but may lack predictive accuracy.^47^ Consequently, potentially more sensitive and specific assessment instruments of early pulmonary health changes are in development.^48^

Biomarkers of potential harm (BOPH), measured in blood and urine, can also be corroborative of risk, as they reflect the impact of exposure to toxicants on downstream physiological systems and thus can have relevant diagnostic sensitivity and specificity. For some diseases which may take decades to develop, biomarkers may also be predictive of future events. Notably, the PATH study captures biomarkers of exposure (BOE), BOPH, and harm outcomes in an integrated longitudinal record, and offers an important data source for ongoing and future research.^49^^,^^50^

#### Definition of incremental risk and risk reduction

In an observational study, non-tobacco users often represent the normative control group for odds adjustments. The control sample should have an adjusted odds ratio (aOR) of 1.0, reflecting the normalized background risk rate due to non–tobacco use sources. These risk sources may include genetic factors, lifestyle and environmental factors such as poor diet and exercise, alcohol, cannabis and other drug use, and exposure to pollution and secondhand smoke.^51^

The aOR observed in people who use tobacco products includes the baseline risk (of 1.0) from non–tobacco use sources, plus incremental excess risk due to tobacco product use (risk in excess of 1.0, see Equations SI.1-2). For example, CC use has been causally linked with increased risk of cardiovascular disease (CVD), stroke, and other respiratory diseases.^52^^,^^53^ Relative risk is the ratio of these excess risks due to product use (Equation SI.3).

**Figure 5:**
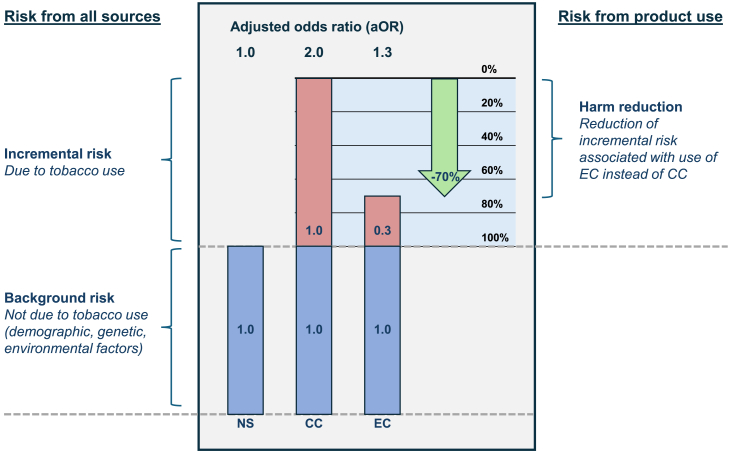
Incremental risk and risk reduction (illustrative example) NS (non-smoker/vaper); CC (combusted cigarette); EC (electronic cigarette). Green arrow represents reduction in risk associated with EC use vs. CC use. In this illustrative hypothetical example, a non–tobacco user (NS) has an adjusted odds ratio (aOR) of 1.0 for a given harm, reflecting the background risk rate. If CC smoking doubles the risk of harm, a subject engaging in CC smoking would have aOR of 2.0. In other words, they would incur a risk of 1.0 from non-tobacco sources, and an additional incremental risk of 1.0 from CC smoking. Likewise, if using EC caused 30% of the incremental risk of CC smoking, use of EC would have aOR of 1.3. This aOR would comprise risk of 1.0 from non-tobacco sources and 0.3 from EC use. In this example, if the subject had used EC instead of smoking CC, their aOR would be 1.3 instead of 2.0. The harm reduction associated with EC would be 70%, due to the incremental risk of 0.3 (i.e., 1.3–1.0) with EC vs. 1.0 for CC (i.e., 2.0–1.0). Note that reduction in all sources of risk is 35% for EC vs. CC users (1.3 vs. 2.0), but harm reduction is 70% (0.3 vs. 1.0 incremental risk due specifically to tobacco product use). See also Equations SI.1-4.

Harm reduction refers to the reduction in the excess risk due to tobacco use, when a lower-risk tobacco product is used instead of a higher-risk tobacco product. The equation describing harm reduction for use of EC compared to CC is derived in Equation SI.4, and an illustrative example is provided in Figure 5.

#### Verification of dose-response

Verification of a dose-response relationship between exposure and outcome is of fundamental importance. Lack of a dose-response relationship should lead to examination of potential counterfactual explanations. A common dose-response comparison is between every day, some day, former, and never users, but as discussed previously, precision may be limited without incorporation of duration of use or cumulative exposures to all products (i.e., pack-years of CC and duration of regular use of EC, or lifetime number of EC uses).

For dual-use populations, biomarker studies suggest that EC use which displaces CC use causes a reduction in exposure to CC toxins.^50^ The counterargument has been made that each type of product presents unique risks and so DU risk is EC risk x CC risk.^54^ Precisely characterizing the exposure history of each product (CC pack-years, and EC use frequency x duration of regular use) within the DU sample in a non-randomized observational study is critical to differentiating between these two hypotheses.

In some non-randomized observational studies, a mixed linear model approach is used to estimate risk associated with EC use and CC use as independent variables, without measuring DU risk directly.^54^ Because of the positively and negatively correlated interactions between EC and CC use, the standard error is subject to bias in dual-users. Consequently, the impact of DU vs. CC use should be directly measured as an independent sample rather than extrapolated to be equal to the impact of EC vs. non-use. At a minimum, the goodness of fit and actual confidence interval for the subsample of dual users must be verified to validate the precision and accuracy of imputation in a mixed linear model. However, the DU, switching and exclusive EC ever-use cohorts should preferably be evaluated individually in a parallel-cohort design.

In retrospective studies using databases such as PATH which do capture timing of initiation of regular use, another verification opportunity could be to confirm that risk was not elevated until after exposure began. For example, in a population which uses EC, aOR of events in the time before starting EC should be 1.0 if covariate adjustments have canceled out all sources of bias. In other words, zero exposure dose should correspond to zero incremental risk.

#### Sufficient sample size and number of harm events

Sample size sufficiency can be a challenge in non-randomized observational studies of tobacco and nicotine exposure. A generally accepted rule of thumb is a harm event per variable ratio (EPV) of 10 to avoid problems parameterizing the models.^55^^,^^56^ Insufficient EPV can introduce bias into regression coefficients and confidence intervals, and may even produce paradoxical results (significance in the wrong direction). It is imperative that researchers carefully evaluate EPVs (see Document S1 for additional details).

#### Comparing cohort samples with minimal demographic overlap

Adjustments of odds ratios are typically derived from a linear fit of a regression model. However, linear fits may introduce confounding if variables are not independent or do not follow linear relationships. One common example is the effect of age, where a linear fit (i.e., adjustment = m∗age + b) may not reflect effects that take years to accumulate and then manifest increasingly rapidly at older ages. If an EC sample is much younger than a control sample, then linear adjustments may result in an under- or overestimate of risk. Likewise, correlation between CC and EC use is higher in older adults (see Document S1) and thus correlation of CC harm may be higher if these interdependencies are not accounted for. One mitigation strategy is to stratify age into deciles, and adjust for each decile independently, or to restrict the analytic sample or perform a sensitivity test on age ranges where chronic disease outcomes are physiologically plausible.^57^^,^^58^ While stratification by age may seemingly reduce analytic power by reducing sample sizes, it may in actuality increase accuracy of odds adjustments because of “apples to apples” comparisons.

#### Transparent peer review and consideration of counterfactuals

The authors of the ROBINS framework suggest that counterfactuals should always be carefully analyzed by subject area specialists and by methodologists. Specifically, this approach stresses the importance of evaluating risks of bias in making causal assessments in all non-randomized studies.^9^ Similarly, including experts spanning a breadth of perspectives across multiple domain areas can help to verify and validate study precision and accuracy.

## A case study highlights opportunities for improved precision and accuracy of observational studies

The ROBINS-informed best-practice considerations outlined above are next discussed in the context of a recent meta-analysis of 107 observational studies (Glantz et al., 2024; see Document S2 for comprehensive details and analysis).^10^ The meta-analysis was designed to determine (1) whether EC are harm-reduced relative to CC as single agents and (2) whether DU of both products is more harmful than exclusive smoking of CC.

### Risk associated with EC vs. CC use

**Figure 6:**
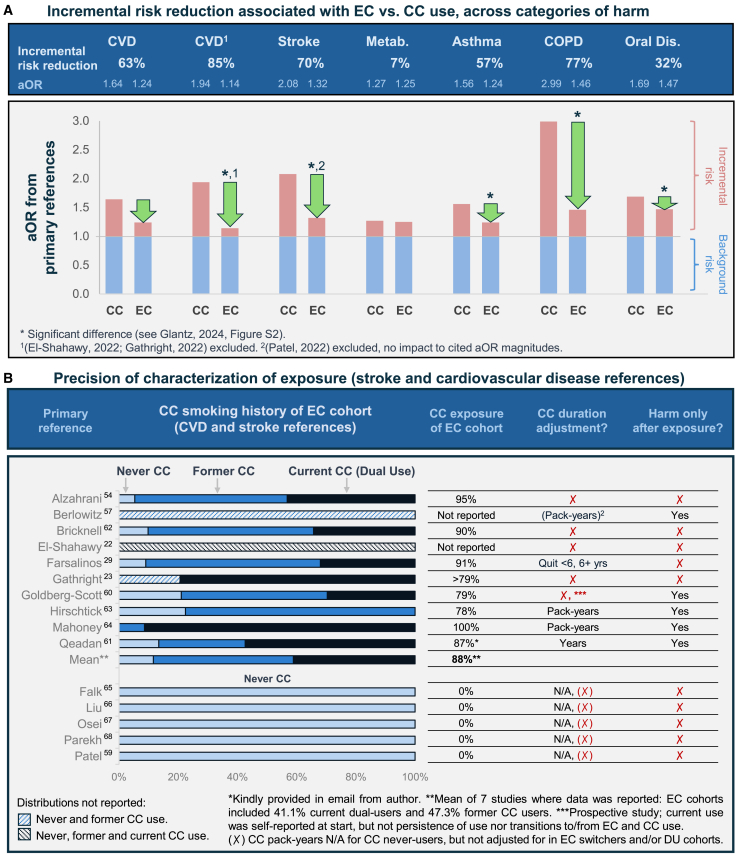
Characterization of risk associated with EC use (case study) aOR (adjusted odds ratio); CC (combusted cigarette); EC (electronic cigarette), Metab. (metabolic disease); Oral Dis. (oral disease). (A) Incremental risk reduction associated with EC vs. CC use across categories of harm. All aOR are cited from (Glantz et al., 2024, Table 1), with the exception of CVD,^1^ which reflects re-analysis after exclusions of two references which segmented into harm vs. no-harm cohorts and reported that CC use was not associated with increased risk for CVD.^22^^,^^23^ (B) Precision of characterization of exposure to CC and EC in CVD and stroke-related references cited by (Glantz et al., 2024).^10^ Leftmost column: primary reference cited. Next column: distribution of CC use histories among the EC cohort (light blue = never CC use; medium blue = former CC use; dark blue = current CC use). Rightmost columns: percentage of EC cohort which formerly smoked or currently smoke CC, whether odds adjustments were made for duration or timing of CC use, and whether harm events occurring before the start of EC use were adjusted for. Red X represents adjustments not made. See also Equations SI.1-4, Figures S2.1–S2.4, and Tables S2.1–S2.8 for more detailed information and data analysis.

In the meta-analysis (Glantz et al., 2024, Figure 1 and Table 1), the authors reported odds for all-source (tobacco and non-tobacco) risk in people who use EC vs. CC.^10^ However, this does not accurately reflect harm reduction. The actual magnitude of tobacco-related risk reduction indicated by these references is now reported here in Figure 6 (panel A) across six disease areas, and ranges from 7% for metabolic disease to 85% for cardiovascular disease (CVD). Risk reduction magnitudes associated with displacing CC use with EC use were calculated by inputting the aORs reported from the primary references into Equation SI.4.

The magnitude of EC and DU risks reported in the primary references incorporated multiple confounds impacting accuracy and precision. As illustration, the meta-analysis included 15 unique studies pertaining to CVD or stroke risk.^22^^,^^23^^,^^29^^,^^54^^,^^57^^,^^59^^,^^60^^,^^61^^,^^62^^,^^63^^,^^64^^,^^65^^,^^66^^,^^67^^,^^68^ The authors concluded that EC are not safer than CC with respect to CVD and stroke but also reported that each of these conclusions was brittle with respect to a single study. Upon inspection, both studies contained major errors or characteristics which should have disqualified them from inclusion. Patel et al., 2022, cited a sample size inconsistent with 2 waves of NHANES (*n* = 266,058, which should be *n* = 18,798), and an improbable aOR confidence interval of [1.15–1.16] after a 6-fold OR adjustment and directionality change from EC being lower risk than CC to higher risk than CC.^59^ Gathright et al., 2020, used a linear regression case-control methodology (harm vs. no-harm cohorts, invalid if EC and CC usage were not independent), and subsequently reported that CC were not associated with increased CVD risk (aOR 0.92), while EC were associated with increased risk (aOR 1.49).^23^ Likewise, El Shahawy et al., 2022, reported aOR of 1.05 and 2.24 for CC and EC, respectively (see Document S2 for details).^22^

Figure 6 (panel A) reflects the aOR directly reported in the primary studies cited by the meta-analysis. However, as shown in Figure 6, panel B, in 10 of the 15 studies, the EC cohort included current and former CC users. In these studies, on average (when reported), 88% of the EC cohort currently or formerly smoked CC. Concerningly, half of these studies of exposure did not adjust for duration and timing of CC exposure, in spite of the preponderance of CC use in the EC cohorts, bringing the precision of odds adjustments into question. Furthermore, 10 studies were retrospective, none of which assessed whether the harm event preceded or followed the start of EC use. 5 studies were prospective; one of them did not evaluate tobacco product use state, (including relapse to CC use) during the prospective period.^60^ Correctly adjusting for the imprecisions outlined in Panel B represents an important research opportunity (see Figure S2.1 and Tables S2.1–S2.5 for more details and data analysis).

### Risk associated with DU vs. CC use

In 10 of the 15 CVD and stroke-related primary citations, CC pack-years were not adjusted for, when comparing the DU and CC cohorts. Furthermore, across all cited references, the meta-analysis authors imputed 55 of 113 data points describing the risk of DU vs. CC use by asserting that the ratio describing the risk associated with EC use vs. non-use was equivalent and substitutable. We now report that these 55 imputed data points provided up to 4-fold higher estimates of incremental DU risk in contradistinction to 58 data points where the data was reported in the original study. In addition, in 37 complete data point sets (aOR available for EC, CC and DU from the primary reference), the incremental risk of (DU vs. CC) when imputed by (EC vs. non-use) had no correlation with the actual values. Thus, the risk associated with dual use, across these studies, remains an important topic for future re-analyses (see Figures S2.1–S2.4 and Tables S2.6–S2.8 for more details and data analysis).

## Discussion

Observational studies of exposure to tobacco and nicotine products constitute an important source of real-world data for characterizing the risk-reduction and induction associated with different patterns of product use, including exclusive use, dual-use, switching and stopping tobacco and nicotine product use. These non-randomized study designs are constructed by study authors, who select the definition of cohorts and comparators, exposure measurements, outcomes, and which odds ratio adjustment factors to consider. Therefore, it is important to carefully and comprehensively evaluate all possible sources of confounding inherent within study designs in order to isolate effects that are specific to the use of the product of interest.

Central themes highlighted in this review are that exposure to multiple products should not be assumed to be independent, and that accurate characterization of the relationship between exposure and outcome requires precise selection of cohorts and comparators and complete characterization of dose, duration, and timing of use and stopping of use of two separate sources of exposure, along with relative timing of outcome. Furthermore, verification of dose-response and event to adjustment variable ratio sufficiency can help to validate results. An evaluation of a recent meta-analysis of 107 studies indicated that in many cases, the precision and accuracy of results may be overstated due to inherent confounding which was not accounted for.

The careful and comprehensive incorporation of principles from the ROBINS framework provides an opportunity for improvement of the accuracy of future studies and the precision of data capture of national tobacco use databases. This can help to inform regulatory science and enhance public health, representing a promising PATH forward.

## Appendix

**Table:** 

**Terms**	

CC	Combusted/conventional cigarette
CDC	US Centers for Disease Control and Prevention
CO	Carbon monoxide; COHb is carboxy-hemoglobin; measures of exposure to combustion
COPD	Chronic obstructive pulmonary disease
CVD	Cardiovascular disease
EC	Electronic cigarette; electronic nicotine delivery system
FDA-CTP	US Food and Drug Administration, Center for Tobacco Products
HTP	Heated tobacco product
NIDA	US National Institute on Drug Abuse
NIH	US National Institutes of Health
NRSE	Non-randomized study of exposure; longitudinal parallel cohort study
NRT	Nicotine replacement therapy such as pharmaceutical nicotine patches and gums
ONP	Oral nicotine products such as consumer nicotine pouches
OR	Odds ratio; aOR is adjusted to normalize for covariate imbalances across cohorts
RCT	Randomized controlled trial

**National Surveys Mentioned**	

ARIC	Atherosclerosis Risk in Communities (NIH)
BRFSS	Behavioral Risk Factor Surveillance System (CDC)
NHANES	National Health and Nutrition Examination Survey (CDC)
NHIS	National Health Interview Survey (CDC)
PATH	Population Assessment of Tobacco and Health (FDA, NIH, NIDA)

## Limitations of the study

Future analyses can continue to improve the precision and accuracy of assessment of the risk-inducing and -reducing effects of nicotine and tobacco products. Some topics, such as assessment of EPV ratio sufficiency (harm events per odds adjustment variable) and its impact on confidence intervals of odds ratio adjustments, were somewhat technical and extended beyond the scope of this review. In analyzing a meta-analysis spanning 107 studies, this review focused most intensively on references relevant to stroke and cardiovascular disease; other disease areas can be explored more completely elsewhere.

## Resource availability

### Lead contact

Requests for further information and resources should be directed to and will be fulfilled by the lead contact, Gal Cohen (gal.cohen@roseresearchcenter.com).

### Materials, data and code availability

Analysis source materials are available as follows: the PATH study data referenced in Figure 4 is synthesized from public-use data files. PATH data is available at: https://www.icpsr.umich.edu/web/NAHDAP/studies/36498 (see also Document S1). Equations derived for calculating risk-induction and risk-reduction are provided in Document S1 (Equations SI.1-4). Data analysis used to evaluate the case study is provided in Document S2. Papers comprising the analytic sample of the case study are available on-line. Several sources require payment to the publisher for access.

## Acknowledgments

The authors thank Drs. Marcus Munafò, Mike Cummings and Jed Rose for comments on the manuscript. The authors also thank Drs. Floe Foxon, Saul Shiffman and David Levy for discussion on early findings preceding the writing of the manuscript. Floe Foxon and G.C. previously jointly evaluated the findings from a study which informed the current research (see Document S2). The authors also thank Dr. Fares Qeadan for kindly sharing information which was mentioned in Figure 6.

S.C.’s research contribution and journal open access fees were supported by the 10.13039/100000054National Cancer Institute of the National Institutes of Health (NIH) and FDA Center for Tobacco Products (CTP) under Award Number 2U54CA229974. The content is solely the responsibility of the authors and does not necessarily represent the official views of the NIH or the Food and Drug Administration. This review was not funded by nor commissioned by any other entities.

## Author contributions

Both authors contributed to conceptualization, methodology, and analysis. G.C. was the lead writer and generated the figures. S.C. contributed to writing, review, and editing.

## Declaration of interests

G.C. is a salaried employee of Rose Research Center (RRC), an independent contract research organization that performs studies pertaining to smoking cessation and tobacco harm reduction. Research support for other projects: National Institute on Drug Abuse; Global Action to End Smoking, Inc. (formerly Foundation for a Smoke-Free World, Inc.), a US nonprofit 501(c)(3) private foundation; Nicotine BRST LLC; JUUL Labs; Altria; Embera Neurotherapeutics, Inc.; Otsuka Pharmaceutical; Swedish Match, Philip Morris International. G.C. was previously a Principal Scientist at JUUL Labs. He also was employed at Nektar Therapeutics, whose pipeline included an inhaled NRT. Stock holdings in Qnovia, a developer of an inhaled NRT, and JUUL Labs. This review was not funded nor commissioned by any of these non-RRC entities.

S.C. has no declarations of interest to declare, beyond acknowledgment of NIH and CTP funding.

## Supplemental information

Document S1. Figure S1.1 and Tables S1.1 and S1.2 Detailed background information.

Document S2. Figures S2.1–S2.4 and Tables S2.1–S2.8 Case study-related primary data analysis (Glantz et al., 2024).^10^
