# Supplementary material for: Observational studies of exposure to tobacco and nicotine products: Best practices for maximizing statistical precision and accuracy
Source: iScience. 2025 Feb 8;28(3):111985. doi: 10.1016/j.isci.2025.111985 (PMC11915159; doi:10.1016/j.isci.2025.111985)
Supplement: Document S1. Figure S1.1 and Tables S1.1 and S1.2 — Detailed background information. [file mmc1.pdf]

iScience, Volume *28*

Supplemental information

Observational studies of exposure to tobacco and nicotine products: Best practices for maximizing statistical precision and accuracy

Gal Cohen and Steven Cook

I. DETAILED BACKGROUND INFORMATION

Risk vs. harm as an outcome measure

This review focuses on outcomes measured as aORs, with the acknowledgement that "relative risk" and "harm reduction" are sometimes conflated with respect to this measure. Risk is the expected rate of harm normalized across a given sample or population. A harm event is often measured as a discrete event which either does or does not occur (although in some cases there may be gradations of severity). Risk is usually a continuous variable which may vary over time. Harm reduction, as a colloquially used term, typically encompasses **relative risk** associated with use of one product vs. another, and **risk reversal** due to stopping use of one product and switching to another (see also Equations SI.1-4).

Notably, the US Tobacco Control Act enumerates the reduction of toxicant exposure, risk and harm as differentiated outcomes which can be validated through FDA review and subsequently included in product labeling of consumer tobacco products.[1] FDA-authorized labeling of consumer tobacco products indicating modified exposure or risk include the following:[2]

- IQOS (heated tobacco product): reduced toxicant exposure vs. cigarettes.
- SNUS (oral nicotine product): reduced risk of mouth cancer, heart disease, lung cancer, stroke, emphysema, and chronic bronchitis vs. cigarettes.
- Snuff (oral nicotine product): reduced risk of lung cancer vs. cigarettes.

Key research questions of observational studies of exposure to tobacco and nicotine products

Table SI.1. Key research questions pertaining to risk for non-randomized observational studies of exposure, related to Graphical Abstract

**Q1.**	What is the incremental risk above baseline associated with use of EC or CC, compared to non-use, and relative to each other?
**Q2.**	Is there a reduction in risk associated with displacement of CC with EC?
**Q3.**	What is the risk associated with dual-use of CC and EC?

To address these research questions, populations representative of several categories of tobacco use histories are commonly utilized: exclusive use of EC use, product switching and former CC use, and dual EC and CC use. It should be noted that since it can be challenging to identify large samples of exclusive adult EC users who never used CC products, "EC cohorts" often include a mix of former and sometimes also current CC use histories, therefore Questions 2 and 3 are sometimes inherent in answering Question 1.

Question 3, characterizing the risk of dual-use, is an extension of Question 1, where both EC and CC are concurrently used, but it can also incorporate question 2, to the extent that CC use is displaced by EC use in the sample studied. Thus, assessment of dual-use relative risk requires particular attention to avoiding potential sources of confounding and statistical bias.

Confounding of EC and CC likelihood and frequency of use

- Likelihood and frequency of use of EC and CC are not independent of one another, due to phenomena including common liability (correlation) and displacement (anti-correlation).

EC and CC likelihood of use are positively correlated

- In the NHIS 2021 survey, current EC users were 2.7x more likely than current EC non-users to be current CC users and 1.8x more likely to be former CC smokers, while 55% less likely to be a never smoker or have smoked less than 100 CC.[3]

Table SI.2: EC and CC likelihood of use are not independent (NHIS, 2021), related to Figure 1

		EC Use History			
		All adults	Current EC user (4.5% of all adults)	Not current EC user (95.5% of all adults)	Odds of CC use history status based on current EC status (unadjusted ratios)
CC Use History	CC current use	11.5%	29.4%	10.7%	**2.7**
	CC former use	22.8%	40.3%	22.0%	**1.8**
	CC never use	65.7%	30.3%	67.4%	**0.45**

NHIS 2021 survey, unweighted data. Across all adults, 11.5% were current CC users, 22.8% were former CC users, and 65.7% were never CC users. For current EC users, these CC use histories represented 29.4%, 40.3%, and 30.3%, of the cohort, respectively. For adults who were not current EC users, the corresponding rates were 10.7%, 22.0%, and 67.4%, respectively. Source data may be accessed here: NHIS (CDC), MMWR (NIH).

- Raw data from the PATH study (Wave 6) is shown below, broken out by age deciles. Note that for current EC users who are 35 or older, almost all are current or former users of CC.

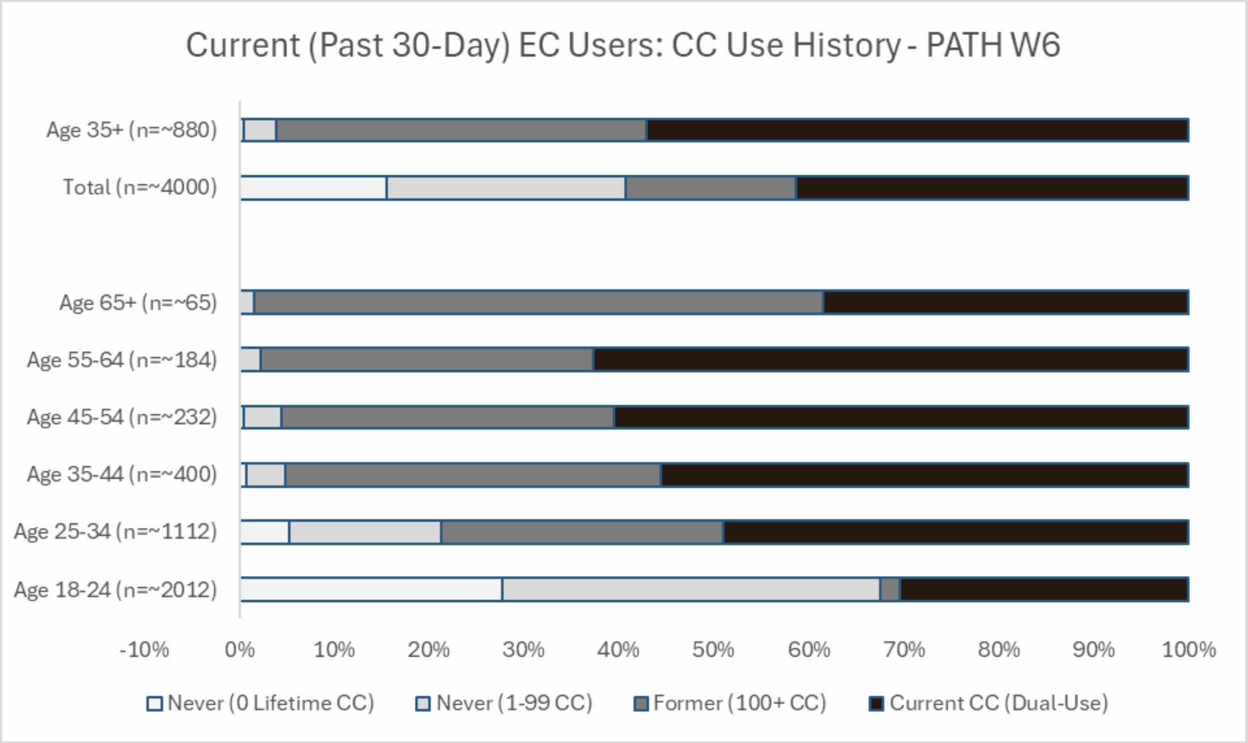

Figure SI.1. CC use history of current EC users (Path Wave 6), related to Figure 4

In this figure, CC use history is shown for six different adult age segments (18-24, 25-34, 35-44, 45-54, 65+). CC use history is segmented into current CC use (with over 100 CC lifetime use), former CC use (over 100+ CC lifetime use), never CC use (1-99 CC lifetime use) and never CC use (0 CC lifetime use). Data are unweighted and reflect the raw data set.

EC and CC frequency of use are anti-correlated

- At the same time, CC quitting is more likely as frequency of use of EC increases.[4–6]

Quantification of risk and risk reduction

- Evaluation of risk involves differentiating between background risks, not associated with tobacco use, and incremental risks associated with tobacco product use. Harm reduction involves the displacement of incremental risk associated with one product with use by another product with a lesser incremental risk impact.

- In an observational study, non-tobacco users often represent the normative control group for odds adjustments. The non tobacco-using control sample should therefore have an adjusted odds ratio (aOR) of 1.0, reflecting the normalized background risk rate due to non-tobacco use sources. These risk sources may include genetic factors, lifestyle and environmental factors such as poor diet and exercise, alcohol, cannabis and other drug use, and exposure to pollution and secondhand smoke.[7]

- The aOR observed in people who use tobacco products includes the baseline risk (of 1.0) from non-tobacco use sources, plus incremental excess risk due to use of the tobacco product (risk in excess of 1.0, see Equations SI.1-2).
    - For example, CC use has been causally linked with increased risk of cardiovascular disease (CVD), stroke, and other respiratory diseases.[8]
    - Relative risk for EC vs. CC is the ratio of these excess risks for EC and CC (see Equation SI.3).

- Harm reduction refers to the reduction in the excess risk due to tobacco use, when a less harmful tobacco product is used instead of a more harmful tobacco product.
    - The equation describing harm reduction for use of EC compared to CC is derived in Equation SI.4, and an illustrative example is provided in Figure 5.

Equations for quantifying relative risk and risk reduction

The equations below quantify the incremental risk-induction and risk-reduction associated with EC and/or CC use. Incremental risk reflects risk specific to tobacco-product use, i.e. risk above the background risk arising from environmental, demographic, and genetic factors.

Equation SI.1. Incremental risk associated with CC use, related to Figures 5-6

Eq. SI.1

$$Incremental\ risk\ associated\ with\ CC\ use$$

$$= ([aOR\ CC] - [aOR\ NS\ Control]) = ([aOR\ CC] - 1.0)$$

Equation SI.2. Incremental risk associated with EC use, related to Figures 5-6

Eq. SI.2

$$Incremental\ risk\ associated\ with\ EC\ use$$

$$= ([aOR\ EC] - [aOR\ NS\ Control]) = ([aOR\ EC] - 1.0)$$

Equation SI.3. Relative risk associated with EC vs. CC use, related to Figures 5-6

Eq. SI.3

$$Relative\ risk\ (associated\ with\ EC\ vs.\ CC\ use) = \frac{EC\ incremental\ risk}{CC\ incremental\ risk} = \frac{(\ [aOR\ EC] - 1.0\ )}{(\ [aOR\ CC] - 1.0)}$$

Equation SI.4. Risk reduction associated with EC displacement of CC use, related to Figures 5-6

Eq. SI.4

$$Harm\ reduction\ (associated\ with\ EC\ displacement\ of\ CC\ use) = \frac{(CC\ incr.risk) - (EC\ inc.risk)}{CC\ incremental\ risk} = 1 - \frac{(\ [aOR\ EC] - 1\ )}{(\ [aOR\ CC] - 1)}$$

Which specific product was used?

The following factors may each impact the risk associated with use of a given EC product (see also Figure 2):

Which active molecule was vaped?

- EC use in some survey questions may encompass cannabis or CBD use, and in other cases is nicotine-free.[9,10]
- Isomer of nicotine. The S-isomer is more potent than the R-isomer. Nicotine extracted from the tobacco plant is ~99% S-nicotine. Synthetic nicotine became widely commercially available recently, and was initially a racemic mix. Currently it is most typically, but not always, purified S-nicotine.
- The impact of exposure to nicotine analogue molecules such as methylated nicotines is also not well understood.[11]
- For nicotine extracted from tobacco, alkaloids such as anabasine and anatabine may be present and may impact P450 metabolism enzymes and nicotinic receptors.[12,13]

E-liquid formulation

- Nicotine can be formulated as a free-base or as nicotine salts with potentially different abuse liability profiles.
- In general, earlier generations of EC utilized lower concentrations of free-base nicotine, while current generations utilize higher concentrations of nicotine salt formulations, with higher nicotine flux. There may be an inverse relationship between nicotine concentration and toxicant exposure due to nicotine titration [14,15]
- Flavorants and impurities may impact emission chemistry and toxicant profile. A wide range of synthetic flavorants and natural extracts appear in different products. Even two products with the same flavor descriptor may have a radically different chemical composition of flavorants.[16]

Aerosol generation and emissions

- The most traditional heating element involves a wick which is wrapped in a metal coil. Some more recent designs utilize a higher capacity ceramic heating element.
- The number of uses per heating element may impact leachables and extractables. For some products, heating elements may be replaced automatically with each pod or cartomizer; in some refillable products, replacement frequency depends on the discretion of the user to swap the coil

out.

- Power and resistance impact heat transferred to the e-liquid and can have dramatic impact on toxicant generation. A widely cited publication showed that high power settings can cause EC to generate higher levels of formaldehyde vs. CC, while typical power settings were associated with dramatically lower levels vs. CC.[17] In many products, power settings are fixed. In mods, settings may vary at the discretion of the user.
- Toxicant exposure may be higher in products which are not temperature regulated.
- Volume: toxicant exposure is related to volume of e-liquid consumed and consequent aerosol inhaled. Newer products with higher nicotine delivery may be associated with increased efficacy in switching and therefore lead to reduced exposure to cigarette toxicants.[6]

Manufacturing, quality, regulatory review, and brand

- FDA authorized products utilize quality system practices and manufacturing processes which pass FDA review. However, most EC products do not apply for FDA authorization, and THC products are not FDA regulated at all. An extreme example was the phenomenon of EVALI-related harm arising from use of Vitamin E as a cost-cutting solubilizing agent by some manufacturers of FDA-unregulated THC-containing vapes.[18]
- Verification of brand and authenticity vs. generic category, unauthorized compatibles and counterfeits.
  - Depending on the specificity of the analysis needed, brand name descriptors, flavor profile, source of purchase, or visual confirmation can all help confirm which product was used, particularly in the case of counterfeits or unauthorized compatible cartomizers and pods.[19]
  - Lastly, “usual product” may or may not be representative of all products used, unless it is confirmed that the usual product was used exclusively.

Product generation

- EC generation most commonly refers to device format (cigalike, tank, mod, pod, disposable)[20]
- However, individual products may also have generations. For instance, the FDA has authorized a second generation (G2) VUSE Solo EC product, but most historic use was of the G1 product.[21]

Biochemical verification of use status

- Propylene glycol (PG) has been proposed as a potential marker for e-cigarette use, however it may not be specific enough to discriminate between EC use vs. non-use, especially in post-2018 observational settings for higher nicotine concentrations.
  - Hiler et al., performed an observational study where the enrolled EC group used 6.5 ml e-liquid per day of 8 mg/ml nicotine, characteristic of older generation tanks and mods (study ran Fall 2016-Spring 2019).[22] Even in that study there were several non-users whose dietary PG intake would have classified them as vapers. A typical contemporary pod user may uses ~0.5ml e-liquid per day of 50-60mg/ml, which would only be 25% of non-EC background, and background SD in non-vapers was 150% of mean levels.[23]
  - Burkhardt et al., performed a confinement study which reported 5ug/ml for EC and 0 for non-EC, but it appears the prescribed diet had no PG, which is not representative of real-world non-EC level of 10ug/ml reported in Hiler.[24] Also, in Burkhardt figure 5A, mean PG consumption was ~1.5g / day. This means participants consumed ~3ml/day of e-liquid (PG content is reported as 50-55%), which again significantly exceeds typical pod consumption. Notably, some of the higher e-liquid consuming users appeared to titrate PG absorption to an asymptote, possibly not inhaling as deeply, which also would reduce signal to noise.

Were sample sizes and number of harm events sufficiently large for model validity?

- A generally accepted rule of thumb is that the EPV ratio (events per variable, e.g. the number of hazard events per odds adjustment regression variable) should be at least 10 for linear regression models to avoid "major problems".[25,26]
- In some cases, 5-9 events per adjustment variable may be sufficient, for instance with use of penalized regression approaches, or when the event fraction (incidence of harm events) is greater than ~10%.[27]
- In other cases, an EPV of 20-50 may be necessary.[28–31]
- A definitive exploration is beyond the scope of this review, but there is a need to more deeply explore this issue. Transparent reporting of the number of hazard events observed in each sample in the raw data, along with the number and magnitude of adjustment variables, can help to verify EPV ratio sufficiency and accuracy of results.

REFERENCES

SI.1. 111th Congress. *Family Smoking Prevention and Tobacco Control Act.*; 2009.

SI.2. Cohen G, Bellanca CM, Bernardini R, Rose JE, Polosa R. Personalized and adaptive interventions for smoking cessation: Emerging trends and determinants of efficacy. *iScience*. Published online October 2024:111090. doi:10.1016/j.isci.2024.111090

SI.3. CDC. QuickStats: Percentage Distribution of Cigarette Smoking Status† Among Current Adult E-Cigarette Users by Age Group — National Health Interview Survey, United States, 2021. *MMWR Morb Mortal Wkly Rep*. 2023;72(10):270. doi:10.15585/mmwr.mm7210a7

SI.4. Wang RJ, Bhadriraju S, Glantz SA. E-Cigarette Use and Adult Cigarette Smoking Cessation: A Meta-Analysis. *Am J Public Health*. 2021;111(2):230-246. doi:10.2105/AJPH.2020.305999

SI.5. Harlow AF, Stokes AC, Brooks DR, et al. Prospective association between e‐cigarette use frequency patterns and cigarette smoking abstinence among adult cigarette smokers in the United States. *Addiction*. 2022;117(12):3129-3139. doi:10.1111/add.16009

SI.6. Kasza KA, Edwards KC, Kimmel HL, et al. Association of e-Cigarette Use With Discontinuation of Cigarette Smoking Among Adult Smokers Who Were Initially Never Planning to Quit. *JAMA Network Open*. 2021;4(12):e2140880. doi:10.1001/jamanetworkopen.2021.40880

SI.7. Martin SS, Aday AW, Almarzooq ZI, et al. 2024 Heart Disease and Stroke Statistics: A Report of US and Global Data From the American Heart Association. *Circulation*. 2024;149(8). doi:10.1161/CIR.0000000000001209

SI.8. Centers for Disease Control and Prevention (US), National Center for Chronic Disease Prevention and Health Promotion (US), Office on Smoking and Health (US). *How Tobacco Smoke Causes Disease: The Biology and Behavioral Basis for Smoking-Attributable Disease: A Report of the Surgeon General*. Centers for Disease Control and Prevention (US); 2010. Accessed April 18, 2017. http://www.ncbi.nlm.nih.gov/books/NBK53017/

SI.9. Bhat TA, Kalathil SG, Goniewicz ML, Hutson A, Thanavala Y. Not all vaping is the same: differential pulmonary effects of vaping cannabidiol versus nicotine. *Thorax*. 2023;78(9):922-932. doi:10.1136/thorax-2022-218743

SI.10. Selya A, Kim S, Shiffman S, Gitchell J, Foxon F. What Substances Are Adolescents Vaping?

Estimating Nicotine-Specific and Cannabis-Specific Vaping from US National Youth Surveys. *Substance Use & Misuse*. 2024;59(2):218-224. doi:10.1080/10826084.2023.2267114

SI.11. Erythropel HC, Jabba SV, Silinski P, et al. Variability in Constituents of E-Cigarette Products Containing Nicotine Analogues. *JAMA*. Published online August 7, 2024. doi:10.1001/jama.2024.12408

SI.12. Jacob P, Chan L, Cheung P, et al. Minor Tobacco Alkaloids as Biomarkers to Distinguish Combusted Tobacco Use From Electronic Nicotine Delivery Systems Use. Two New Analytical Methods. *Front Chem*. 2022;10:749089. doi:10.3389/fchem.2022.749089

SI.13. Denton TT, Zhang X, Cashman JR. Nicotine-related alkaloids and metabolites as inhibitors of human cytochrome P-450 2A6. *Biochemical Pharmacology*. 2004;67(4):751-756. doi:10.1016/j.bcp.2003.10.022

SI.14. El Hourani M, Shihadeh A, Talih S, Eissenberg T. Comparison of Nicotine Emissions Rate, “Nicotine Flux”, from Heated, Electronic, and Combustible Tobacco Products. *Tob Control*. Published online January 27, 2022:tobaccocontrol-2021-056850. doi:10.1136/tobaccocontrol-2021-056850

SI.15. Felicione NJ, Kaiser L, Leigh NJ, et al. Comparing POD and MOD ENDS Users’ Product Characteristics, Use Behaviors, and Nicotine Exposure. *Nicotine & Tobacco Research*. 2023;25(3):498-504. doi:10.1093/ntr/ntac211

SI.16. Kassem NOF, Strongin RM, Stroup AM, et al. A Review of the Toxicity of Ingredients in e-Cigarettes, Including Those Ingredients Having the FDA’s “Generally Recognized as Safe (GRAS)” Regulatory Status for Use in Food. *Nicotine and Tobacco Research*. 2024;26(11):1445-1454. doi:10.1093/ntr/ntae123

SI.17. Jensen RP, Luo W, Pankow JF, Strongin RM, Peyton DH. Hidden Formaldehyde in E-Cigarette Aerosols. *N Engl J Med*. 2015;372(4):392-394. doi:10.1056/NEJMc1413069

SI.18. Marrocco A, Singh D, Christiani DC, Demokritou P. E-cigarette vaping associated acute lung injury (EVALI): state of science and future research needs. *Critical Reviews in Toxicology*. 2022;52(3):188-220. doi:10.1080/10408444.2022.2082918

SI.19. Dell LG, Page MK, Leigh NJ, Goniewicz ML. Removal of mango-flavoured Juul pods created opportunity for adulterated mango Juul-compatible pods with altered chemical constituents. *Tob Control*. 2022;31(Suppl 3):s230-s233. doi:10.1136/tc-2022-057476

SI.20. Smith M, Hilton S. Global Regulatory Approaches towards E-Cigarettes, Key Arguments, and Approaches Pursued. In: Michaud A, P. Stawicki S, Izurieta R, eds. *Sustainable Development*. Vol 17. IntechOpen; 2024. doi:10.5772/intechopen.107343

SI.21. Campbell C, Jin T, Round EK, Schmidt E, Nelson P, Baxter S. Part one: abuse liability of Vuse Solo (G2) electronic nicotine delivery system relative to combustible cigarettes and nicotine gum. *Sci Rep*. 2022;12(1):22080. doi:10.1038/s41598-022-26417-2

SI.22. Hiler M, Breland A, Wolf CE, Poklis JL, Nanco CR, Eissenberg T. Are Urine Propylene Glycol or Vegetable Glycerin Markers of E-cigarette Use or Abstinence? *tob regul sci*. 2020;6(4):235-241. doi:10.18001/TRS.6.4.2

SI.23. Giberson J, Nardone N, Addo N, et al. Nicotine Intake in Adult Pod E-cigarette Users: Impact of User and Device Characteristics. *Nicotine and Tobacco Research*. 2023;25(8):1489-1495. doi:10.1093/ntr/ntad050

SI.24. Burkhardt T, Pluym N, Scherer G, Scherer M. 1,2-Propylene Glycol: A Biomarker of Exposure Specific to e-Cigarette Consumption. *Separations*. 2021;8(10):180. doi:10.3390/separations8100180

SI.25. Peduzzi P, Concato J, Feinstein AR, Holford TR. Importance of events per independent variable in proportional hazards regression analysis II. Accuracy and precision of regression estimates. *Journal of Clinical Epidemiology*. 1995;48(12):1503-1510. doi:10.1016/0895-4356(95)00048-8

SI.26. Peduzzi P, Concato J, Kemper E, Holford TR, Feinstein AR. A simulation study of the number of events per variable in logistic regression analysis. *Journal of Clinical Epidemiology*. 1996;49(12):1373-1379. doi:10.1016/S0895-4356(96)00236-3

SI.27. Lu M, Zhong W, Liu Y, Miao H, Li Y, Ji M. Sample Size for Assessing Agreement between Two Methods of Measurement by Bland-Altman Method. *The International Journal of Biostatistics*. 2016;12(2):8p. doi:10.1515/ijb-2015-0039

SI.28. Austin PC, Steyerberg EW. Events per variable (EPV) and the relative performance of different strategies for estimating the out-of-sample validity of logistic regression models. *Stat Methods Med Res*. 2017;26(2):796-808. doi:10.1177/0962280214558972

SI.29. Pavlou M, Ambler G, Seaman SR, et al. How to develop a more accurate risk prediction model when there are few events. *BMJ*. Published online August 11, 2015:h3868. doi:10.1136/bmj.h3868

SI.30. Van Der Ploeg T, Austin PC, Steyerberg EW. Modern modelling techniques are data hungry: a simulation study for predicting dichotomous endpoints. *BMC Med Res Methodol*. 2014;14(1):137. doi:10.1186/1471-2288-14-137

SI.31. Vittinghoff E, McCulloch CE. Relaxing the Rule of Ten Events per Variable in Logistic and Cox Regression. *American Journal of Epidemiology*. 2007;165(6):710-718. doi:10.1093/aje/kwk052
