# Supplementary material for: Observational studies of exposure to tobacco and nicotine products: Best practices for maximizing statistical precision and accuracy
Source: iScience. 2025 Feb 8;28(3):111985. doi: 10.1016/j.isci.2025.111985 (PMC11915159; doi:10.1016/j.isci.2025.111985)
Supplement: Document S2. Figures S2.1–S2.4 and Tables S2.1–S2.8 — Case study-related primary data analysis (Glantz et al., 2024).10 [file mmc2.pdf]

II. CASE STUDY-RELATED PRIMARY DATA (Glantz et al., 2024)[1]

This recent meta-analysis of 124 citations spanning 107 unique studies was designed to determine 1) whether e-cigarettes (EC) are harm-reduced relative to combusted cigarettes (CC) as single agents, and 2) whether dual-use (DU) of both products is more harmful than exclusive smoking of CC (Glantz et al., 2024).[1] In this Supplement, the meta-analysis results are reviewed in detail, with a specific focus on the impact of EC and CC on cardiovascular disease (CVD) and stroke risk.

Definition and magnitude of relative risk associated with EC vs. CC use

A. Glantz et al., had reported odds for all-source (tobacco use and non tobacco-use) risks in people who use EC vs. CC in Figure 1 of the meta-analysis. This ratio is not an accurate measure of harm reduction (see Table SII.1).
B. The actual magnitude of risk reduction associated in displacing CC use with EC use indicated by these references is now reported here in Figure 6, and Tables SII.1,4 and 5.
C. Furthermore, the magnitude of EC risk is often subject to methodological inaccuracies and imprecisions in the primary references. To examine this, one must step through each study. This Supplement II therefore focuses on the citations for two disease areas, cardiovascular disease (CVD) and stroke.

CVD and stroke references: risk associated with EC vs. CC use

A. The meta-analysis included 18 citations (spanning 15 unique references) pertaining to CVD or stroke risk.
B. The authors concluded that, as single agents, EC are not safer than CC with respect to CVD and stroke. The data from Table 1 of Glantz et al., shows that EC trended as 63% risk-reduced for CVD and 70% risk-reduced for stroke. Wide confidence intervals rendered these results non-significant.
C. Glantz et al., Figure S5 showed that the non-significance result was brittle for CVD and stroke due to one outlier publication for each (El Shahawy et al., 2022; Patel et al., 2022).[2,3]
D. Of the 15 references for stroke and CVD, 3 contained major errors or characteristics which should have disqualified them from inclusion (El-Shahawy et al., 2022; Gathright et al., 2020; Patel et al., 2022).[2–4]
E. Re-analysis with exclusion of these 3 studies found that these citations actually indicate that EC are 85% risk-reduced vs. CC with regards to CVD, and 66% or 70% risk-reduced for stroke (depending on data table used).
F. Furthermore, the studies had significant analytic limitations impacting precision and accuracy, including several which may have lead to an overestimate of EC risk.

CVD and stroke references: risk associated with DU (EC+CC) vs. CC use

A. The authors imputed 55 of 113 data points describing the risk of DU vs. exclusive CC use using the equation (OR DU) / (OR CC) = (OR EC). In other words, the OR of EC vs. non-use (mean value and confidence interval) were directly substituted to represent the OR of DU vs. CC.
B. These 55 imputed data points were found to provide up to four-fold higher estimates of incremental DU risk in contradistinction to 58 data points where the data was reported in the original study.
C. Furthermore, in 37 complete data point sets (aOR available for EC, CC and DU) which were reported in primary references, the incremental OR impact of DU when imputed had no correlation with the actual values.
D. Lastly, these primary references typically did not adjust for duration and intensity of CC and EU use in the DU cohort.

DEFINITION AND MAGNITUDE OF RELATIVE RISK ASSOCIATED WITH EC VS. CC USE

A. The meta-analysis had reported odds for all-source (tobacco and non-tobacco) harms in people who use EC vs. CC. This is not an accurate measure of harm reduction.

Glantz et al., Figure 1 reports (aOR EC) / (aOR CC), which is the ratio of all-source (tobacco and non-tobacco) risk for users of EC vs. CC.[1]

Glantz et al., Table 1 also reports this:

- For CVD, (aOR EC)/(aOR CC) = 1.24/1.64 = 0.76, which approximates 0.81 after meta-analysis model adjustments
- For stroke, (aOR EC)/(aOR CC) = 1.32 / 2.08 = 0.66 which approximates 0.73 after meta-analysis model adjustments

Table SII.1. Pooled aOR from (Glantz et al., 2024, Table 1),[1] related to Figure 6

	Disease Area					
	CVD*	Stroke	Metabolic Dysfunction	Asthma	COPD	Oral Disease
**Comparison to cigarette use (EC vs. CC)**						
**aOR: EC vs. CC mean, [CI]**	**0.81 [0.58-1.14]**	**0.73 [0.47-1.13]**	**0.99 [0.91-1.09]**	**0.84 [0.75-0.95]**	**0.53 [0.38-0.74]**	**0.87 [0.76-1.00]**
**Comparison to no use (NU)**						
**aOR: EC vs. NU**	**1.24**	**1.32**	**1.25**	**1.24**	**1.46**	**1.47**
**aOR: CC vs. NU**	**1.64**	**2.08**	**1.27**	**1.56**	**2.99**	**1.69**
**Implied reduction in incremental risk associated with use of EC instead of CC**						
**Risk Reduction**	**63%**	**70%**	**7%**	**57%**	**23%**	**32%**

Abbreviations: CVD (cardiovascular disease); aOR (adjusted odds ratio); EC (electronic cigarette); CC (combusted cigarette); NU (non-use of tobacco and nicotine products)

B. The actual magnitude of risk reduction associated in displacing CC use with EC use indicated by these references is now reported here.

Relative risk associated with EC and CC product use was not calculated in Glantz et al., and Is larger than indicated

- Glantz et al., explicitly claim that (aOR EC)/(aOR CC) is a direct indicator of magnitude of harm reduction.

  > "Although lower than for cigarettes, the reduced odds ratios associated with e-cigarette use compared with cigarettes for asthma (odds ratio, 0.84), COPD (odds ratio, 0.53), and oral disease (odds ratio, 0.87) are 3 to 10 times the 15% risk that the FDA or the 5% risk that Public Health England have quoted."[1]

- However, as discussed earlier, the authors are inappropriately conflating harm reduction with reduction in risk from all sources. It would be a very surprising result if e-cigarettes reduced the harm caused by non-tobacco sources such as alcohol, cars, industrial pollution, fireplaces, forest fires, poor diet and exercise, along with genetic factors. When the FDA or Public Health England describe harm reduction, they are specifically referring to the reduction in incremental risk above

baseline caused by e-cigarette use vs. cigarettes, as defined in Equation SI.4. This harm reduction metric was not reported in the meta-analysis.

- See Figure 6 and Tables SII.1,4,5 for calculation of risk induction and reduction associated with CC and EC use indicated by the references cited by the meta-analysis.

C. Furthermore, the magnitude of EC risk is often subject to methodological inaccuracies and imprecisions in the primary references. To examine this, one must step through each study. This Supplement II therefore focuses on the citations for two disease areas, cardiovascular disease (CVD) and stroke.

CVD AND STROKE REFERENCES: RISK ASSOCIATED WITH EC vs. CC USE

A. The meta-analysis included 18 citations (spanning 15 unique references) pertaining to CVD or stroke risk.

To better understand the precision and accuracy of the 124 citations in the meta-analysis requires reading each of the references closely. As a representative subset more tractable to readers of this review, the 15 studies (18 citations) comprising the references for CVD and stroke were analyzed in detail; readers are encouraged to read these 15 primary references directly.

Description of referenced set of 18 citations

- 18 citations were referenced for stroke and CVD, spanning 15 unique publications
- The referenced studies used several different approaches (see Tables SII.2-3)
  - Five different primary databases utilized
  - Prospective vs. retrospective
  - Cohort selection (EC vs. CC; case control).
    Case control refers to hazard vs. no-hazard segmentation.
  - Inclusion of former and current smoking in EC cohort

Table SII.2. Source databases for primary references incorporated in (Glantz et al., 2024)[1] meta-analysis pertaining to CVD or stroke, related to Figure 6

Database	Year	Study
PATH	2013-19	Berlowitz (2022)[5]
PATH	2016-18	El-Shahawy (2022)[2]
PATH	2016-17	Farsalinos (2019)[6]
PATH	2013-14	Gathright (2019)[4]
PATH	2013-19	Hirschtick (2022)[7]
PATH	2022	Mahoney (2022)[8]
PATH	2014-18	Qeadan (2023)[9]
NHIS	2014-16	Alzharani (2018)[10]

NHIS	2014, 2016-18	Falk (2022)[11]
BRFSS	2020	Liu (2022)[12]
BRFSS	2016-17	Osei (2019a)[13]
BRFSS	2016	Bricknell (2021)[14]
BRFSS	2016-17	Parekh (2020a)[15]
NHANES	2015-18	Patel (2022)[3]
Kaiser Permanente Research Bank	2015-19	Goldberg-Scott (2023)[16]

Table SII.3. Design characteristics of CVD and stroke primary references of (Glantz et al., 2024),[1] related to Figure 6

**Temporal design**	Prospective	Retrospective		
**Cohort selection**	EC vs. CC	EC vs. CC	EC vs. CC	Case Control (Hazard / no hazard)
**EC cohort includes former / current CC?**	Includes	Excludes	Includes	Includes
**CVD Citations**	• Berlowitz<br>• Goldberg-Scott<br>• Hirschtick<br>• Mahoney<br>• Qeadan	• Falk<br>• Liu<br>• Osei	• Alzahrani<br>• Farsalinos	• El-Shahawy<br>• Gathright
**Stroke Citations**	• Goldberg-Scott<br>• Hirschtick	• Falk<br>• Parekh<br>• Patel	• Bricknell	

B. The authors concluded that, as single agents, EC are not safer than CC with respect to CVD and stroke.

- The data from (Glantz et al.,Table 1)[1] show that EC trended as risk reduced for CVD and for stroke. Wide confidence intervals rendered these results as non-significant. The EC vs. CC aOR ratio (for all-source risk) for cardiovascular disease was 0.81 [0.58-1.14] and for stroke was 0.73 [0.47-1.13] (see Table SII.1).
- The authors concluded, “Direct epidemiological evidence based on actual use of e-cigarettes in the general population suggests that, at least for cardiovascular disease, stroke, and metabolic dysfunction, the odds of disease between current e-cigarette and cigarette use were similar.”[1]

C. **The authors demonstrated that each of these stroke and CVD non-significance results were brittle with respect to a single outlier paper.**

- Stroke:
    - See (Glantz et al., Figure S5, “Stroke” panel),[1] showing brittleness with respect to Patel citation.[3] This citation changes a major result of the meta-analysis; its inclusion changes EC from being significantly less harmful vs. CC for stroke, to being statistically not different.
- CVD:
    - See (Glantz et al., Figure S5, “Cardiovascular” panel).[1]
    - The El-Shahawy citation is shown to change a major result of the meta-analysis: its inclusion changes EC from being significantly less harmful vs. CC for CVD, to being statistically not different.[2]
    - The Gathright citation is shown to further amplify the brittleness of this result.

D. **Of the 18 citations for stroke and CVD, 3 contained major errors or characteristics which should have disqualified them from inclusion (El-Shahawy et al., 2022; Gathright et al., 2020; Patel et al., 2022).[2–4]**

Stroke citations: (Patel et al., 2022)[3]

- This paper was submitted May 1, 2022 and published May 27, 2022
- Impossible sample size reported from NHANES (n=266,058 for 2 waves 2015-2018). Correct sample size should be n=18,798.
    - NHANES 2015-16: n=9,544.[17]
    - NHANES 2017-18: n=9,254.[18]
- EC/CC confidence interval (CI) of 0.00 (see Glantz et al., Figure 1)[1] is improbably precise after 6-fold adjustment in OR -> aOR and change in polarity from less harmful than CC to more harmful than CC.
    - Reported stroke prevalence (unadjusted) was 6.75% for CC vs. 1.09% for EC.
    - This was adjusted to aOR (EC/CC) = 1.15 [1.15,1.16] in the primary reference.
- The finding that strokes happened at a younger age in the EC group was likely a trivial consequence of the EC sample being younger to begin with than the comparison sample:
    - Onset of stroke was 11 years earlier for EC
        - EC users age of onset = 48 years old
        - CC users age of onset = 59 years old
    - The authors of the primary reference have declined multiple requests to provide the underlying age distribution of the EC group vs. the CC group, in conflict with their data and analysis sharing agreement in the paper.
- Duration of EC and CC use was not reported, only current use status (past 30 days). It was not reported what percent of EC users were former CC users.
- It was not confirmed whether stroke events happened before or after the start of EC usage.
- Questions about this study have also been discussed in the journal Science.[19]

Cardiovascular disease citations: (El-Shahawy et al., 2022; Gathright et al., 2019)[2,4]

- The El-Shahawy and Gathright references used a case-control approach (segmentation into harm vs. no-harm cohorts), which requires that EC and CC use be independent and thus is highly susceptible to confounding. These studies both found that combusted cigarettes were safe, while e-cigarettes were harmful
- El-Shahawy et al., 2022
    - Endpoint of erectile dysfunction (ED)
    - CC use was reported as having no impact (aOR = 1.05)
    - Lack of effect of CC use on ED was not in line with previous studies:
        - "The odds ratio of smokers with ED has ranged between 1.4 and 3.1 with statistically significant confidence intervals in the vast majority of (other) studies."[20]
        - Former CC use was reported as trending towards being protective (aOR = 0.84), and represented half of the comparator sample.
        - Raw OR of 1.51 was adjusted to aOR of 0.84.
    - EC daily use aOR 2.24 was reported.
        - OR 0.72 was adjusted to aOR 2.24
        - Authors did report a dose-response: aOR 1.43 for current EC some-day users and 1.12 for EC former users.
    - Temporality is also a concern (events occurring before start of EC use were not censored)
    - A letter to the editor raised concerns about use of a single ED self-report question; the authors replied that they believed the question was valid and overall ED prevalence was "in line with national surveys".[21,22]

- Gathright et al., 2019
    - This study analyzed the PATH Wave 1 dataset (2013). In this time period, due to the novelty of EC products, most adults with EC history had limited duration of EC use and were former or current CC users.
    - The study explored self-report of congestive heart failure diagnosis, but did not confirm whether the diagnosis happened before or after start of use of EC. The study did not measure duration and intensity of CC and EC use.
    - Former CC use was also not tracked nor adjusted for.
    - In the unweighted data set, EC use was highly confounded with CC use.
        - 2,878 of 3,620 (80%) of current EC users were also dual-using CC.
        - In contrast, 2,878 of 13,337 (22%) of current CC users were also dual-using EC
        - In dual-using participants with heart failure, 90% endorsed using EC as a means of reducing their CC use, vs. 30% for those without heart failure, highlighting the complex interdependence between these variables and reverse causality (heart failure diagnosis causes increased of likelihood of subsequent EC use).
    - In the regression analysis of the case-control segmentation (harm vs. no-harm):
        - CC use trended as protective vs. HF (aOR 0.92).
        - EC use had aOR of 1.49.
        - DU use had aOR of 1.76.

- The finding that CC use is not associated with HF is not in line with the literature, which consistently shows a statistically significant impact of CC use on HF. For instance, a meta-analysis of prospective studies of adults 18+ reported that current CC use was associated with OR of 1.6 for HF.[23]
- Lastly, exclusive current use of EC appeared to trend as protective in the weighted stratified data, which was not discussed in the paper. Of 70 harm events that occurred in current users of ECs, 60 occurred in dual-users, and only n=10 in exclusive current CC users, further impacting validity of regression analysis to distinguish between the impact of EC vs. CC.

E. Re-analysis with exclusion of these 3 studies found that these citations actually indicate that EC are 85% risk-reduced vs. CC with regards to CVD, and 66% or 70% risk-reduced for stroke (depending on data table used).

EC vs. CC relative risk for stroke

- Table SII.4 indicates that relative incremental risk associated with EC use is 66% less than the risk associated with EC use, reflecting aOR from (Glantz et al., 2024, Figures S2 and S4).[1]
- This approximates the 70% reduction in risk indicated by Table SII.1 and Figure 6, both of which reflect aOR reported in (Glantz et al., 2024, Table 1).[1]

Table SII.4. OR reported from stroke references cited by (Glantz et al., 2024),[1] related to Figure 6

Glantz et al., Citation #	Glantz et al., Reference	Fig 1 EC/CC	Fig S2 EC	Fig S4 CC	Risk Reduction
13	Bricknell (2021)	0.77	1.62	2.10	
14	Falk (2022)	0.50	1.06	2.11	
**15**	Goldberg Scott (2023)	(N/A)	1.65	(N/A)	
16	Hirschtick (2022)	0.77	1.74	2.26	
17	Parekh (2020a)	0.43	0.69	1.59	
**18**	***Patel (2022)***	***1.15***	***(N/A)***	***(N/A)***	
**Average of all citations**		**0.73**	**1.35**	**2.02**	**66%**
**Average of all citations (excluding ref 18)**		**0.62**	**1.35**	**2.02**	**66%**

EC vs. CC relative risk for cardiovascular disease

- Table SII.5 indicates that relative incremental risk associated with EC use is 63% less than the risk associated with EC use, reflecting aOR from (Glantz et al., 2024, Figures S2 and S4).[1]
- This mirrors the 63% reduction in risk indicated by Table SII.1 and Figure 6, both of which reflect aOR reported in (Glantz et al., 2024, Table 1).[1]
- With exclusion of (El-Shahawy, 2022 and Gathright, 2019)[2,4] for using an invalid case-control approach (which showed no risk associated with CC use), risk reduction is 85%.

Table SII.5. OR reported from CVD references cited by (Glantz et al., 2024),[1] related to Figure 6

Glantz et al., Citation #	Glantz et al., Reference	Fig 1 EC/CC	Fig S2 EC	Fig S4 CC	Risk Reduction
1	Alzahrani (2018)	0.66	1.79	2.72 (2.29, 3.24)	
2	Berlowitz (2022)	0.66	1.00	1.53 (1.26, 1.86)	
**3**	***El-Shahawy (2022)***	***2.13***	***2.24***	***1.05 (0.68, 1.62)***	
4	Farsalinos (2019)	0.76	1.31	1.73 (1.46, 2.05)	
5	Falk (2022)	0.35	0.98	2.84 (2.47, 3.27)	
**6**	***Gathright (2019)***	***1.62***	***1.49***	***0.92 (0.75, 1.13)***	
7	Goldberg Scott (2023)	(N/A)	1.30	(N/A)	
8	Hirschtick (2022)	0.30	0.61	1.99 (1.29, 3.07)	
9	Liu (2022)	0.87	1.17	1.35 (1.20, 1.51)	
10	Mahoney (2022)	(N/A)	(NA)	1.44 (0.78, 2.67)	
11	Osei (2019a)	(N/A)	1.04	(N/A)	
12	Qeadan (2023)	(N/A)	1.02	(N/A)	
**Average of all citations**		**0.92**	**1.27**	**1.73**	**63%**
**Average (excluding refs 3&6)**		**0.60**	**1.14**	**1.94**	**85%**

F. Furthermore, the other studies had significant analytic limitations impacting accuracy and precision, including several which may have lead to an overestimate of EC risk.

As shown in Figures 6 and SII.1, these issues include:

- EC cohort inclusion of current or former CC use without precise adjustments:
  - Current / former CC cohorts not used as controls
  - Duration of CC exposure not adjusted for
  - Time quit not adjusted for in CC to EC switchers vs. CC former users
- Retrospective studies: ensuring that exposure preceded harm
- Prospective studies: verifying continuity or transition of use state

Figure SII.1. Issues impacting precision and accuracy of CVD and stroke references**,** related to Figure 6

Limitations in references for CVD and stroke

Temporal Design	Prospective	Retrospective		
Cohorts	EC vs. CC	EC vs. CC	EC vs. CC	Case-Control (Hazard vs. No Hazard)
EC Includes Former and Current CC Use?	Includes	Excludes*	Includes	Includes
CVD Citations	• Berlowitz<br>• Goldberg-Scott (C) (D)<br>• Hirschtick<br>• Mahoney<br>• Qeadan	• Falk (T) (D)<br>• Liu (T) (D)<br>• Osei (T) (D)	• Alzharani (T) (D)<br>• Farsalinos (T)	• El-Shahawy (T) (D)<br>• Gathright (T) (D)
Stroke Citations	• *Goldberg-Scott* (C) (D)<br>• *Hirschtick*	• *Falk* (T) (D)<br>• Parekh (T) (D)<br>• Patel (T) (D)	• Bricknell (T) (D)	

(T) Temporal order: harm could precede exposure
(D) Duration of CC smoking or time quit not factored in
*These studies segmented out CC current users who were CC never users (CC adjustment N/A) vs. CC former or current users.
(C) Continuity / transitions of use state not verified

Six citations did not sufficiently factor in total tobacco use history for the EC cohort (and an additional five did not adjust for it in their dual-use cohorts).

- In 12 citations (spanning 10 studies), the EC use population included former and current CC use; this represented ~88% of the EC population in the 9 citations (spanning 7 studies) in which these percentages were provided, with former CC smoking the predominant use pattern.
- In these EC cohorts with so few never CC smokers, how the CC use history was adjusted for is highly critical for the reliability of the study. And yet, in 6 of those citations (spanning 5 studies), no adjustment was made to balance time/duration of CC use.
- Note that this includes the Goldberg-Scott reference, which was cited 5 times in total in the meta-analysis, representing the most cited reference (Goldberg Scott et al., 2023).[16] In this study, intensity and duration of CC use and time since quitting smoking in former CC users were not reported nor adjusted for, in spite of CC smoking reported as being 40x more likely in the current EC group. Additionally, the study population was queried once about current EC use status (use at least once in the past 30 days preceding study start) and then prospectively followed for an average of 45 months without confirmation of persistence of use state nor whether CC relapse occurred in any of the cohorts. Furthermore, raw age distributions were highly unbalanced across cohorts. Geographic sampling region introduced imbalances of 2x in the OR. This database is proprietary to Kaiser-Permanente and no public use files were available for independent verification.
- An additional six citations (from five unique) studies segmented out EC users who were CC never users, but in their evaluation of EC users who were former or current CC users, they did not adjust for CC use duration.

11 citations for CVD and Stroke included harm events which occurred before start of EC use

- 11 citations spanning 10 unique studies did not adjust for harm events which occurred before the start of EC exposure.
- The temporality issue was raised in a letter to the editor re: Alzharani.[6,10] In a response, the authors asserted that the issue could be dismissed because results were "what one would expect."[24] This was also discussed in another publication.[25]

DU (EC + CC) vs. EXCLUSIVE CC USE

A. The authors imputed 55 of 113 data points describing the risk of DU vs. exclusive CC use using the equation (OR DU) / (OR CC) = (OR EC). In other words, the OR of EC vs. non-use (mean value and confidence interval) were directly substituted to represent the OR of DU vs. CC.

Imputation of relative risk of DU vs. CC use by substitution of EC vs. NU aOR ratio in 55 cases

- The authors asserted that DU odds ratios can be precisely and accurately imputed from EC and CC odds ratios, by multiplying odds ratios (DU = EC*CC).
    - In other words, the incremental risk of DU vs. CC has the same value as the incremental risk of EC vs. non-tobacco use, OR (DU/CC) = OR EC.
    - Furthermore the same confidence intervals were used for (DU vs. CC) as for (EC vs. non-tobacco use).
    - In Table SII.6, the rightmost 2 columns have equivalent values indicating substitution.

Table SII.6. Listing of imputed data points for DU/CC OR in (Glantz et al., 2024),[1] related to Figure 6

Type of Harm	Ref #	Author	Year	Glantz et al., Figure 2 DU/CC aOR	Glantz et al., Figure S2 EC aOR
CVD	1	Alzahrani	2018	1.79 (1.20, 2.67)	1.79 (1.20, 2.67)
CVD	3	El-Shahawy	2022	2.24 (1.42, 3.54)	2.24 (1.42, 3.54)
CVD	4	Farsalinos	2019	1.31 (0.79, 2.17)	1.31 (0.79, 2.17)
CVD	7	Goldberg Scott	2023	1.30 (0.66, 2.56)	1.30 (0.66, 2.56)
CVD	12	Qeadan	2023	1.02 (0.89, 1.17)	1.02 (0.89, 1.17)
CVD		IMPUTED (N=5)		1.53 *(mean)*	
Stroke	13	Bricknell	2021	1.62 (1.16, 2.27)	1.62 (1.16, 2.27)
Stroke	15	Goldberg Scott	2023	1.65 (0.94, 2.89)	1.65 (0.94, 2.89)
Stroke		IMPUTED (N=2)		1.64	
Metab	24	Kim	2020b	1.40 (1.08, 1.81)	1.40 (1.08, 1.81)
Metab	29	Sompa	2022	1.90 (1.03, 3.50)	1.90 (1.03, 3.50)
Metab	30	Zhang	2022	1.22 (1.09, 1.36)	1.22 (1.09, 1.36)
Metab		IMPUTED (N=3)		1.51	
Asthma	32	Bayly	2019	0.90 (0.71, 1.15)	0.90 (0.71, 1.15)
Asthma	33	Bhatta	2020	1.30 (0.79, 2.13)	1.30 (0.79, 2.13)
Asthma	35	Boyd	2021	1.09 (0.75, 1.58)	1.09 (0.75, 1.58)
Asthma	36	Braymiller	2020	0.85 (0.54, 1.34)	0.85 (0.54, 1.34)

Asthma	37	Brunette	2023	1.12 (0.45, 2.82)	1.12 (0.45, 2.82)
Asthma	38	Chaffee	2021a	1.36 (0.95, 1.95)	1.36 (0.95, 1.95)
Asthma	40	Choi	2016	1.78 (1.2, 2.64)	1.78 (1.2, 1.64)
Asthma	43	Han	2020	1.31 (1.09, 1.58)	1.31 (1.09, 1.58)
Asthma	44	Kim	2017	1.13 (1.01, 1.26)	1.13 (1.01, 1.26)
Asthma	45	Lee	2023	1.22 (1.04, 1.43)	1.22 (1.04, 1.43)
Asthma	48	McConnell	2017	1.24 (0.78, 1.98)	1.24 (0.78, 1.98)
Asthma	49	Osei	2019b	1.39 (1.08, 1.78)	1.39 (1.08, 1.78)
Asthma	56	Schweitzer	2017	1.48 (1.24, 1.71)	1.48 (1.24, 1.77)
Asthma	59	Tackett	2020	1.35 (0.49, 3.75)	1.35 (0.49, 3.75)
Asthma	60	Tackett	2023	1.55 (1.18, 2.04)	1.55 (1.18, 2.04)
Asthma	61	Tanski	2022	1.25 (0.75, 2.09)	1.25 (0.75, 2.09)
Asthma	62	To	2023	1.21 (0.95, 1.54)	1.21 (0.95, 1.54)
Asthma	63	Tran	2020	1.04 (0.92, 1.17)	1.04 (0.92, 1.17)
Asthma	64	Varella	2022	1.41 (0.85, 2.34)	1.41 (0.85, 2.34)
Asthma	65	Walker	2021	1.06 (0.4, 2.81)	1.06 (0.4, 2.81)
Asthma	70	Wills	2022	1.20 (1.06, 1.35)	1.20 (1.06, 1.35)
Asthma	71	Xie	2020b	1.32 (0.92, 1.89)	1.32 (0.92, 1.89)
Asthma		IMPUTED (N=22)		1.25	
COPD	74	Barrameda	2021	1.83 (1.59, 2.1)	1.83 (1.59, 2.1)
COPD	75	Bhatta	2020	1.44 (0.79, 2.62)	1.44 (0.79, 2.62)
COPD	77	Cook	2023b	1.10 (0.74, 1.64)	1.10 (0.74, 1.64)
COPD	80	Goldberg Scott	2023	0.96 (0.71, 1.3)	0.96 (0.71, 1.3)
COPD	86	Perez	2019b	1.43 (1.07, 1.91)	1.43 (1.07, 1.91)
COPD	87	Qeadan	2023	1.11 (0.97, 1.27)	1.11 (0.97, 1.27)
COPD	88	Strong	2018	1.39 (1.06, 1.83)	1.39 (1.06, 1.83)
COPD	90	Wills	2022	1.44 (1.21, 1.71)	1.44 (1.21, 1.71)
COPD	92	Xie	2020b	1.57 (1.08, 2.29)	1.57 (1.08, 2.29)
COPD		IMPUTED (N=9)		1.36	
Oral	95	Atuegwu	2019b	1.58 (1.06, 2.35)	1.58 (1.06, 2.35)
Oral	96	Chaffee	2021b	1.40 (0.69, 2.84)	1.40 (0.69, 2.84)
Oral	98	Cho	2017	1.00 (0.71, 1.4)	1.00 (0.71, 1.4)
Oral	99	Huilgol	2019	1.78 (1.38, 2.29)	1.78 (1.38, 2.29)
Oral	100	Jeong	2020	2.33 (1.58, 3.44)	2.33 (1.58, 3.44)
Oral	101	Silveira	2022	1.15 (0.89, 1.48)	1.15 (0.89, 1.48)
Oral		IMPUTED (N=6)		1.54	
Other	104	Regan	2021	1.09 (0.85, 1.4)	1.09 (0.85, 1.4)
Other	107	McBride	2021	1.59 (1.06, 2.38)	1.59 (1.06, 2.38)
Other	111	Goldberg Scott	2023	1.17 (1.05, 1.3)	1.17 (1.05, 1.3)
Other	115	Wiener	2020	1.82 (1.18, 2.8)	1.82 (1.18, 2.8)
Other	117	Smith	2023	1.35 (1.16, 1.58)	1.35 (1.16, 1.58)
Other	118	Agoons	2021	1.43 (0.84, 2.44)	1.43 (0.84, 2.44)
Other	119	Goldberg Scott	2023	0.80 (0.41, 1.56)	0.80 (0.41, 1.56)
Other		IMPUTED (N=8)		(N/A)	

For 58 of 113 citations, reported value from primary reference was used as the value for DU / CC ratio in meta-analysis (i.e. value was not imputed from EC aOR).

- In Table SII.7, the rightmost 2 columns have different values indicating that they were measured separately by the primary authors, and not substituted for each other.

Table SII.7. Listing of DU/CC aOR data points actually reported in primary references cited by (Glantz et al., 2024),[1] related to Figure 6

Type of Harm	Ref #	Author	Year	Figure 2 DU/CC	Figure S2 EC aOR
CVD	2	Berlowitz	2022	1.01 (0.77, 1.32)	1.00 (0.64, 1.57)
CVD	5	Falk	2022	1.35 (1.08, 1.69)	0.98 (0.55, 1.73)
CVD	6	Gathright	2019	1.91 (1.25, 2.92)	1.49 (0.77, 2.88)
CVD	8	Hirschtick	2022	0.93 (0.28, 3.07)	0.61 (0.08, 4.39)
CVD	9	Liu	2022	0.47 (0.35, 0.63)	1.17 (0.97, 1.41)
CVD	10	Mahoney	2022	1.28 (0.38, 4.35)	(NA)
CVD	11	Osei	2019a	1.36 (1.18, 1.56)	1.04 (0.63, 1.72)
CVD		ACTUALLY REPORTED (N=7)		1.19	
Stroke	14	Falk	2022	1.14 (0.90, 1.43)	1.06 (0.71, 1.58)
Stroke	16	Hirschtick	2022	0.50 (0.15, 1.67)	1.74 (0.55, 5.47)
Stroke	17	Parekh	2020a	1.83 (0.98, 3.42)	0.69 (0.30, 1.56)
Stroke	18	Patel	2022	1.14 (1.14, 1.14)	(NA)
Stroke		ACTUALLY REPORTED (N=4)		1.15	
Metab	20	Cai	2023	1.21 (0.97, 1.50)	0.75 (0.34, 1.64)
Metab	21	Cook	2023a	0.95 (0.70, 1.29)	1.00 (0.68, 1.47)
Metab	22	Falk	2022	1.20 (1.07, 1.35)	1.24 (1.04, 1.47)
Metab	23	Kim	2020a	1.57 (1.03, 2.40)	(NA)
Metab	26	Miller	2021	1.30 (0.99, 1.71)	1.31 (1.05, 1.63)
Metab	27	Okafor	2022	1.05 (0.47, 2.34)	1.73 (0.83, 3.61)
Metab	28	Shi	2022	1.04 (0.65, 1.67)	1.51 (0.93, 2.46)
Metab		ACTUALLY REPORTED (N=7)		1.19	
Asthma	31	Alnajem	2020	1.11 (0.56, 2.20)	1.85 (1.02, 3.37)
Asthma	39	Cho	2016	1.30 (0.86, 1.96)	2.74 (1.3, 5.78)
Asthma	41	Chung	2020	0.75 (0.42, 1.33)	(NA)
Asthma	42	Cordova	2022	1.00 (0.68, 1.47)	0.80 (0.57, 1.13)
Asthma	46	Li	2020	1.03 (0.88, 1.2)	1.68 (1.32, 2.14)
Asthma	47	Mattingly	2023	0.72 (0.27, 1.93)	1.50 (0.83, 2.72)
Asthma	50	Parekh	2020b	2.11 (1.61, 2.76)	1.74 (1.17, 2.58)
Asthma	51	Patel	2023	0.92 (0.44, 1.93)	1.25 (0.69, 2.27)
Asthma	53	Reddy	2021	1.24 (0.92, 1.67)	1.17 (0.69, 1.99)
Asthma	54	Sargent	2022	0.91 (0.61, 1.36)	1.05 (0.61, 1.81)
Asthma	55	Schneller	2020	0.39 (0.11, 1.41)	1.44 (0.93, 2.23)
Asthma	57	Sompa	2022	2.25 (0.83, 6.11)	1.20 (0.34, 4.27)
Asthma	66	Wang	2016	1.15 (0.81, 1.63)	1.28 (1.06, 1.55)
Asthma	67	Williams	2023	0.86 (0.35, 2.12)	1.12 (0.97, 1.29)
Asthma	68	Wills	2019	0.99 (0.8, 1.22)	1.27 (0.96, 1.68)
Asthma	69	Wills	2020	1.30 (1.08, 1.57)	1.29 (1.04, 1.59)
Asthma	72	Xie	2022	0.91 (0.63, 1.32)	1.32 (1.01, 1.73)
Asthma		ACTUALLY REPORTED (N=17)		1.11	
COPD	73	Antwi	2022	0.99 (0.63, 1.55)	1.53 (1.08, 2.16)
COPD	81	Hedman	2018	1.580*	1.46 (0.93, 2.29)
COPD	82	Kim	2021	1.25 (0.69, 2.27)	(NA)
COPD	83	Osei	2020	1.66 (1.46, 1.89)	1.75 (1.14, 2.69)
COPD	84	Parekh	2020b	1.55 (1, 2.4)	1.37 (0.59, 3.16)
COPD	85	Paulin	2022	1.04 (0.74, 1.46)	1.36 (0.48, 3.85)

COPD	89	Wills	2019	1.32 (0.98, 1.77)	2.58 (1.36, 4.89)
COPD	91	Xie	2020a	1.16 (1.03, 1.31)	1.47 (0.92, 2.36)
COPD		ACTUALLY REPORTED (N=8)		1.32	
Oral	93	Akinkube	2019	1.15 (0.77, 1.72)	1.11 (0.79, 1.55)
Oral	94	AlQobaly	2022	1.38 (0.97, 1.97)	0.95 (0.24, 3.79)
Oral	97	Chaffee	2022	1.02 (0.78, 1.35)	1.28 (0.93, 1.76)
Oral		ACTUALLY REPORTED (N=3)		1.18	
Other	103	Hawkins	2021	0.80 (0.55, 1.17)	1.39 (0.84, 2.3)
Other	105	Wang	2020	1.29 (0.92, 1.82)	1.2 (0.52, 2.79)
Other	106	Wen	2023	0.93 (0.75, 1.15)	0.99 (0.78, 1.26)
Other	109	Gaiha	2020	4.47 (0.62, 32.05)	1.91 (0.77, 4.73)
Other	110	Moyers	2023	1.20 (0.74, 1.94)	1.30 (1.01, 1.68)
Other	112	To	2023	1.24 (0.8, 1.92)	1.73 (1, 3)
Other	113	Zhu	2023	1.29 (0.94, 1.76)	0.84 (0.52, 1.36)
Other	114	Christian	2023	1.11 (0.91, 1.36)	0.99 (0.61, 1.6)
Other	116	Tian	2022	1.55 (1.42, 1.69)	1.25 (1, 1.57)
Other	120	Xie	2020c	1.39 (1.08, 1.79)	1.96 (1.16, 3.31)
Other	121	Han	2023	1.20 (0.86, 1.69)	(NA)
Other	122	Wang	2022	1.39 (1.23, 1.58)	1.62 (1.18, 2.23)
Other		ACTUALLY REPORTED (N=12)		(N/A)	

*Hedman data point was a typo in Glantz et al., Fig 2, in which it incorrectly reflected DU, not DU/CC (=1.58).

B. These 55 imputed data points were found to provide up to four-fold higher estimates of incremental DU risk in contradistinction to 58 data points where the data was reported in the original study.

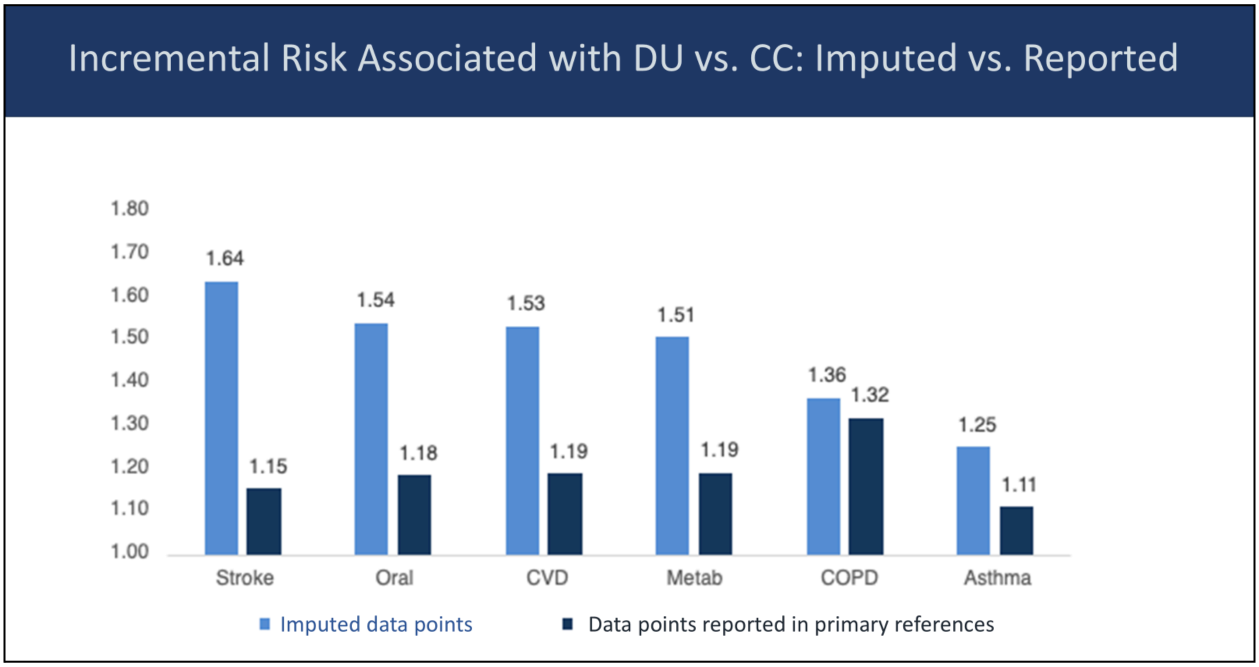

Figure SII.2. Disease-specific risks associated with DU vs. CC use: imputed vs. reported data points, related to Figure 6

Light blue bars: data points imputed by authors of meta-analysis (n=55). Dark blue bars: data points reported in primary references (n=58).

C. **Furthermore, in 37 complete data point sets (aOR available for EC, CC and DU) which were reported in primary references, the incremental OR impact of DU when imputed had no correlation with the actual values.**

- The validity of substituting EC/NU to impute DU/EC was previously asserted in the Alzharani reference, which had common authors with the meta-analysis (Alzahrani et al., 2018).
- However this approach is only valid if there is complete independence between EC and CC use, and if the underlying covariates are similar under DU and non-DU conditions.
- The meta-analysis asserts that (aOR EC)*(aOR CC) can be used to predict and substitute for (OR DU).
    - (aOR DU) = (aOR EC)*(aOR CC)
- In other words, the incremental increase in aOR for (DU vs. CC) is aOR EC (see also Appendix)
    - (aOR DU) / (aOR CC) = (aOR EC)

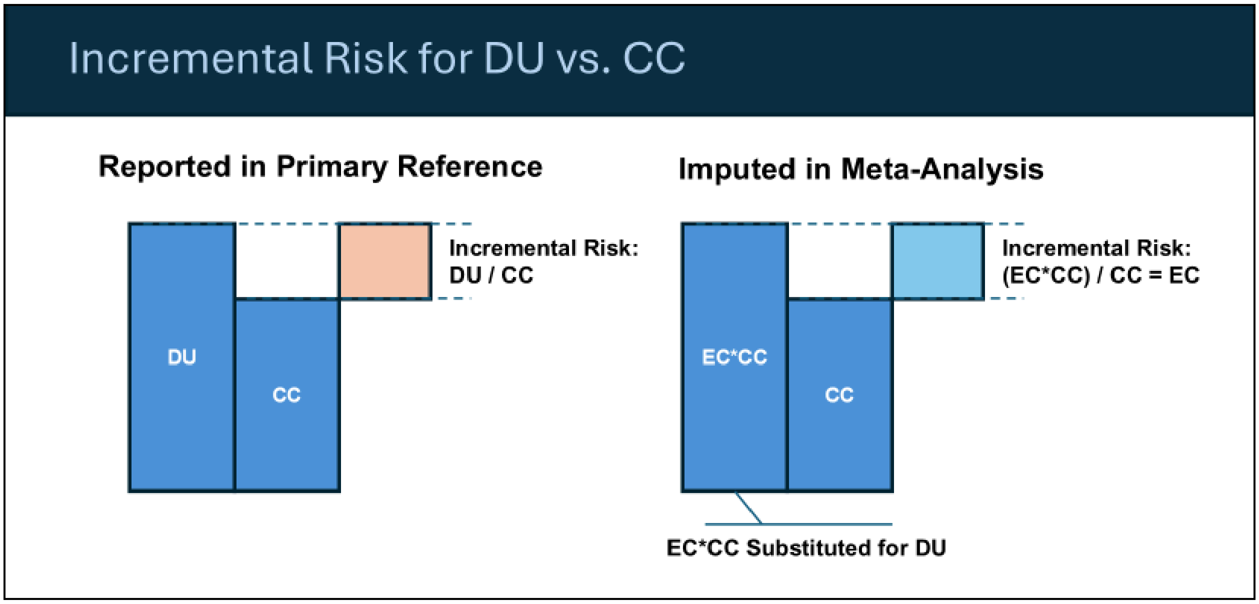

Figure SII.3. Illustration of actual incremental risk and imputation method for quantifying incremental risk for DU vs. CC use. related to Figure 6

The left panel indicates that when both DU and CC aOR are reported in the primary reference, the incremental impact (DU vs. CC) can be found directly. The right panel represents an approach where the incremental impact is imputed by multiplying aOR EC*CC to derive DU.

The imputation method was tested in a separate set of references

- For DU/CC = EC to have been valid, would have required proving that EC and CC are independent and linear, and for all covariates to be adjusted for, across the 55 references in which this approach was applied.
- EC aOR (in cases where former and current CC use are not segmented out) does not minimize MSE (mean square error) for dual use, but rather provides one value for three different conditions of EC use: DU, former CC use, and never CC use.

- To test the validity of this approach, one may assess 37 citations (spanning 34 unique studies) in the meta-analysis in which samples were stratified into CC vs. EC vs. DU, and aOR were reported for all 3.
- As shown in Figure SII.4, actual incremental impact of (DU vs. CC, Y axis) shows negligible correlation (R^2=.04) with the incremental impact of (EC vs. NU, X axis)

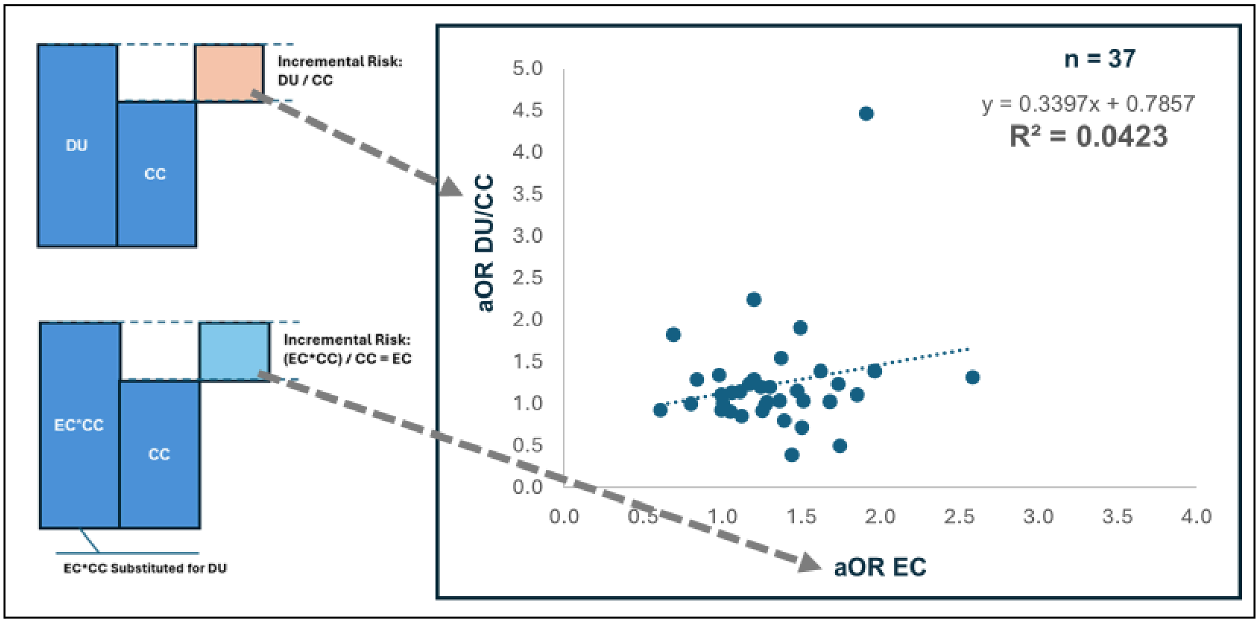

Figure SII.4: Plot of (aOR DU/CC) vs. (aOR EC) from primary references which provided complete data set of aOR EC,CC and DU, related to Figure 6
X-axis: incremental impact of EC vs. non-use. Y-axis: incremental impact of DU vs. CC use.

Table SII.8. Data used to generate Figure SII.4, related to Figure 6

Harm	Cite #	Author	Year	Y-AXIS Fig. SII.4<br>aOR DU/CC (Glantz et al. Fig 2)	X-AXIS Fig. SII.4<br>aOR EC (Glantz et al., Fig S2)	aOR DU (FigS3)	aOR CC (Fig S4)
CVD	2	Berlowitz	2022	1.01	1.00	1.54	1.53
CVD	5	Falk	2022	1.35	0.98	3.84	2.84
CVD	6	Gathright	2019	1.91	1.49	1.76	0.92
CVD	8	Hirschtick	2022	0.93	0.61	1.84	1.99
Stroke	14	Falk	2022	1.14	1.06	2.40	2.11
Stroke	16	Hirschtick	2022	0.50	1.74	1.12	2.26
Stroke	17	Parekh	2020a	1.83	0.69	2.91	1.59
Metab	21	Cook	2023a	0.95	1.00	1.15	1.21
Metab	22	Falk	2022	1.20	1.24	1.66	1.38
Metab	28	Shi	2022	1.04	1.51	1.45	1.39
Asthma	31	Alnajem	2020	1.11	1.85	1.92	1.73
Asthma	42	Cordova	2022	1.00	0.80	0.80	0.80
Asthma	46	Li	2020	1.03	1.68	2.83	2.75

Asthma	47	Mattingly	2023	0.72	1.50	1.23	1.71
Asthma	51	Patel	2023	0.92	1.25	1.54	1.68
Asthma	53	Reddy	2021	1.24	1.17	2.22	1.78
Asthma	54	Sargent	2022	0.91	1.05	2.13	2.34
Asthma	55	Schneller	2020	0.39	1.44	1.52	3.93
Asthma	57	Sompa	2022	2.25	1.20	3.60	1.60
Asthma	67	Williams	2023	0.86	1.12	0.68	0.79
Asthma	68	Wills	2019	0.99	1.27	1.26	1.27
COPD	84	Parekh	2020b	1.55	1.37	5.07	3.28
COPD	85	Paulin	2022	1.04	1.36	1.99	1.92
COPD	89	Wills	2019	1.32	2.58	3.92	2.98
COPD	91	Xie	2020a	1.16	1.47	4.39	3.80
Oral	93	Akinkube	2019	1.15	1.11	1.72	1.50
Oral	97	Chaffee	2022	1.02	1.28	1.80	1.76
Other	103	Hawkins	2021	0.80	1.39	1.03	1.28
Other	105	Wang	2020	1.29	1.20	2.07	1.60
Other	106	Wen	2023	0.93	0.99	1.18	1.26
Other	109	Gaiha	2020	4.47	1.91	6.84	1.53
Other	110	Moyers	2023	1.20	1.30	0.77	0.64
Other	112	To	2023	1.24	1.73	2.13	1.72
Other	113	Zhu	2023	1.29	0.84	1.78	1.38
Other	114	Christian	2023	1.11	0.99	1.28	1.15
Other	120	Xie	2020c	1.39	1.96	2.07	1.49
Other	122	Wang	2022	1.39	1.62	1.84	1.33

D. Lastly, these primary references typically did not adjust for duration and intensity of CC and EC use in the DU cohort.

- See prior section (impact of EC vs. CC, Figure SII.1) and Figure 6 for discussion of this issue. Briefly, a non-randomized study of exposure needs to comprehensively quantify exposure to be accurate.

References

SII.1. Glantz SA, Nguyen N, Oliveira Da Silva AL. Population-Based Disease Odds for E-Cigarettes and Dual Use versus Cigarettes. *NEJM Evidence*. 2024;3(3). doi:10.1056/EVIDoa2300229

SII.2. El-Shahawy O, Shah T, Obisesan OH, et al. Association of E-Cigarettes With Erectile Dysfunction: The Population Assessment of Tobacco and Health Study. *American Journal of Preventive Medicine*. 2022;62(1):26-38. doi:10.1016/j.amepre.2021.08.004

SII.3. Patel U, Patel N, Khurana M, et al. Effect Comparison of E-Cigarette and Traditional Smoking and Association with Stroke—A Cross-Sectional Study of NHANES. *Neurology International*. 2022;14(2):441-452. doi:10.3390/neurolint14020037

SII.4. Gathright EC, Wu WC, Scott-Sheldon LAJ. Electronic cigarette use among heart failure patients: Findings from the Population Assessment of Tobacco and Health study (Wave 1: 2013–2014). *Heart & Lung*. 2020;49(3):229-232. doi:10.1016/j.hrtlng.2019.11.006

SII.5. Berlowitz JB, Xie W, Harlow AF, et al. E-Cigarette Use and Risk of Cardiovascular Disease: A Longitudinal Analysis of the PATH Study (2013–2019). *Circulation*. 2022;145(20):1557-1559. doi:10.1161/CIRCULATIONAHA.121.057369

SII.6. Farsalinos K, Niaura R. E-cigarette Use and Myocardial Infarction: Association Versus Causal Inference. *American Journal of Preventive Medicine*. 2019;56(4):626-627. doi:10.1016/j.amepre.2018.11.013

SII.7. Hirschtick JL, Cook S, Patel A, et al. Longitudinal Associations Between Exclusive and Dual Use of Electronic Nicotine Delivery Systems and Cigarettes and Self-Reported Incident Diagnosed Cardiovascular Disease Among Adults. *Nicotine & Tobacco Research*. 2023;25(3):386-394. doi:10.1093/ntr/ntac182

SII.8. Mahoney MC, Rivard C, Kimmel HL, et al. Cardiovascular Outcomes among Combustible-Tobacco and Electronic Nicotine Delivery System (ENDS) Users in Waves 1 through 5 of the Population Assessment of Tobacco and Health (PATH) Study,

2013–2019. *IJERPH*. 2022;19(7):4137. doi:10.3390/ijerph19074137

SII.9. Qeadan F, Nicolson A, Barbeau WA, Azagba S, English K. The association between dual use of electronic nicotine products and illicit drugs with adverse cardiovascular and respiratory outcomes in a longitudinal analysis using the Population Assessment of Tobacco and Health (PATH) survey. *Drug and Alcohol Dependence Reports*. 2023;7:100166. doi:10.1016/j.dadr.2023.100166

SII.10. Alzahrani T, Pena I, Temesgen N, Glantz SA. Association Between Electronic Cigarette Use and Myocardial Infarction. *American Journal of Preventive Medicine*. 2018;55(4):455-461. doi:10.1016/j.amepre.2018.05.004

SII.11. Falk GE, Hayrettin Okut, Ph.D., Mohinder R. Vindhyal, M.D., M.Ed., Elizabeth Ablah, Ph.D., Mph. Hypertension and Cardiovascular Diseases among Electronic and Combustible Cigarette Users. *kjm*. 2022;15(2):226-230. doi:10.17161/kjm.vol15.16752

SII.12. Liu X, Yuan Z, Ji Y. The association between electronic cigarettes, sleep duration, and the adverse cardiovascular outcomes: Findings from behavioral risk factor surveillance system, 2020. *Front Cardiovasc Med*. 2022;9:909383. doi:10.3389/fcvm.2022.909383

SII.13. Osei AD, Mirbolouk M, Orimoloye OA, et al. Association Between E-Cigarette Use and Cardiovascular Disease Among Never and Current Combustible-Cigarette Smokers. *The American Journal of Medicine*. 2019;132(8):949-954.e2. doi:10.1016/j.amjmed.2019.02.016

SII.14. Bricknell RAT, Ducaud C, Figueroa A, et al. An association between electronic nicotine delivery systems use and a history of stroke using the 2016 behavioral risk factor surveillance system. *Medicine*. 2021;100(36):e27180. doi:10.1097/MD.0000000000027180

SII.15. Parekh T, Pemmasani S, Desai R. Risk of Stroke With E-Cigarette and Combustible Cigarette Use in Young Adults. *American Journal of Preventive Medicine*. 2020;58(3):446-452. doi:10.1016/j.amepre.2019.10.008

SII.16. Goldberg Scott S, Feigelson HS, Powers JD, et al. Demographic, Clinical, and Behavioral Factors Associated With Electronic Nicotine Delivery Systems Use in a Large Cohort in the United States. *Tob Use Insights*. 2023;16:1179173X221134855. doi:10.1177/1179173X221134855

SII.17. CDC. NHANES Analytic Guidelines.pdf. National Health and Nutrition Examination Survey: Analytic Guidelines, 2011-2014 and 2015-2016. 2018. Accessed October 21, 2024. https://wwwn.cdc.gov/nchs/data/nhanes/analyticguidelines/11-16-analytic-guidelines.pdf

SII.18. US Dept. of Health and Human Services, ODPHP. National Health and Nutrition Examination Survey. Published online 2024. https://odphp.health.gov/healthypeople/objectives-and-data/data-sources-and-methods/data-sources/national-health-and-nutrition-examination-survey-nhanes

SII.19. Joelving F. Prescription for controversy. *Science*. Published online 2024. doi:10.1126/science.adq2869

SII.20. Kovac JR, Labbate C, Ramasamy R, Tang D, Lipshultz LI. Effects of cigarette smoking on erectile dysfunction. *Andrologia*. 2015;47(10):1087-1092. doi:10.1111/and.12393

SII.21. El Shahawy O, Loney T, Shah T, Sherman SE, Blaha MJ. Response to Letter Regarding the Article “Association of E-Cigarettes With Erectile Dysfunction: The Population Assessment of Tobacco and Health Study.” *American Journal of Preventive Medicine*. 2022;63(3):e105-e106. doi:10.1016/j.amepre.2022.03.031

SII.22. Capodicasa G, Caponnetto P, Polosa R, Calogero AE. “Association of E-Cigarettes With Erectile Dysfunction: The Population Assessment of Tobacco and Health Study.” *American Journal of Preventive Medicine*. 2022;63(3):e103-e104. doi:10.1016/j.amepre.2022.03.030

SII.23. Lee H, Son, Y-J. “Influence of Smoking Status on Risk of Incident Heart Failure: A Systematic Review and Meta-Analysis of Prospective Cohort Studies.” *Int. J. Environ.*

Res. Public Health. 2019;*16*(15), 2697. doi:10.3390/ijerph16152697.

SII.24. Alzahrani T, Glantz SA. The Association Between E-cigarette Use and Myocardial Infarction Is What One Would Expect Based on the Biological and Clinical Evidence. *American Journal of Preventive Medicine*. 2019;56(4):627. doi:10.1016/j.amepre.2018.11.006

SII.25. Rodu B, Plurphanswat N. Cross-sectional e-cigarette studies are unreliable without timing of exposure and disease diagnosis. *Intern Emerg Med*. 2023;18(1):319-323. doi:10.1007/s11739-022-03141-3

APPENDIX

Contextual excerpts from (Glantz et al., 2024, Supplement)[1]

Definitions of acronyms

EM "Ecig risk (multivariate)"
ES "Ecig risk (stratified)"
CM "Cig risk (multivariate)"
CS "Cig risk (stratified)"
DNM "Dual vs nothing (multivariate)"
DNS "Dual vs nothing (stratified)"
DSM "Dual vs smoking (multivariate)"
DSS "Dual vs smoking (stratified)"
ECM "Ecig vs cig (multivariate)"
ECS "Ecig vs cig (stratified)"
EN "Ecig vs nothing among never smokers (stratified)"
EN "Ecig vs nothing among former smokers (stratified)" (typo, meant EF)

From Supplement, pp 101-102 (Glantz et al., 2024)[1]

DU vs. CC calculation: using EC risk as an estimate ("EM")

"/*Dual vs cig comparison
In the multivariate models, that include only ecgis and cigs, the ecig vs. nothing risk is also the dual vs. cigs risk because eicgs and cigs have independent effects. Specifically, the ecig risk is also the marginal risk above smoking.

"As a result, we have three different estimates of the dual vs. cig comparison:

1. Estimate using the cig vs. nothing risk as an estimate of the dual vs. cig comparison (EM)
2. Direct estimates from multivariate models (DSM)
3. Direct estimates from stratified models (DSS)
4. Calculate estimate from stratified dual vs. nothing (DNS) and cig vs. nothing (CNS) (added to accommodate Chaffee (2022) (DSSSC)

Because there are a few studies in which we have multiple estimates, we will pick the estimate with the smallest point estimates"

"1. Alzahrani (2018) EM = 1.79 …. DC = 1.79; Marginal multivariate marginal ecig risk
3. El-Shahawy (2022) EM = 2.24 … DC = 2.24; Marginal multivariate marginal ecig risk
4. Farsalinos (2019) EM = 1.31 … DC = 1.31; Marginal multivariate marginal ecig risk
7. Goldberg Scott (2023) EM = 1.3 … DC = 1.3; Marginal multivariate marginal ecig risk
12. Qeaden (2023) EM = 1.02 … DC = 1.02; Marginal multivariate marginal ecig risk"
etc.

From (Glantz et al., 2024, Supplement, pp 108-111).[1]
Yellow highlight added for emphasis.
